# Non-Typical Fluorescence Effects and Biological Activity in Selected 1,3,4-thiadiazole Derivatives: Spectroscopic and Theoretical Studies on Substituent, Molecular Aggregation, and pH Effects

**DOI:** 10.3390/ijms20215494

**Published:** 2019-11-04

**Authors:** Iwona Budziak, Dariusz Karcz, Marcin Makowski, Kamila Rachwał, Karolina Starzak, Alicja Matwijczuk, Beata Myśliwa-Kurdziel, Anna Oniszczuk, Maciej Combrzyński, Anna Podleśna, Arkadiusz Matwijczuk

**Affiliations:** 1 Department of Chemistry, University of Life Sciences in Lublin, 20-950 Lublin, Poland; iwona.budziak@up.lublin.pl; 2 Department of Analytical Chemistry (C1), Faculty of Chemical Engineering and Technology, Cracow University of Technology, Warszawska 24, 31-155 Cracow, Poland; dkarcz@chemia.pk.edu.pl (D.K.); kstarzak@chemia.pk.edu.pl (K.S.); 3 Department of Theoretical Chemistry, Faculty of Chemistry, Jagiellonian University, Gronostajowa 2, 30-387 Krakow, Poland; makowskm@chemia.uj.edu.pl; 4 Department of Biotechnology, Microbiology and Human Nutrition, University of Life Sciences in Lublin, Skromna 8, 20-704 Lublin, Poland; kamila.rachwal@up.lublin.pl; 5 Department of Biophysics, University of Life Sciences in Lublin, Akademicka 13, 20-950 Lublin, Poland; alicja.matwijczuk@up.lublin.pl; 6 Department of Plant Physiology and Biochemistry, Faculty of Biochemistry, Biophysics and Biotechnology, Jagiellonian University, 30-387 Krakow, Poland; b.mysliwa-kurdziel@uj.edu.pl; 7 Department of Inorganic Chemistry, Medical University in Lublin, 20-059 Lublin, Poland; anna.oniszczuk@umlub.pl; 8 Department of Thermal Technology and Food Process Engineering, University of Life Sciences in Lublin, 20-950 Lublin, Poland; maciej.combrzynski@up.lublin.pl; 9 Department of Plant Nutrition and Fertilization, Institute of Soil Science and Plant Cultivation-State Research Institute, 24-100 Pulawy, Poland; ap@iung.pulawy.pl

**Keywords:** molecular aggregation, 1,3,4-thiadiazole, molecular spectroscopy, dual fluorescence effects, effect of the substituent group structure

## Abstract

The below article presents the results of spectroscopic research, theoretical (time-dependent density functional theory (TD-DFT)), microbiological, and antioxidative calculations for three compounds from the group of 1,3,4-thiadiazoles: 2-amino-5-phenyl-1,3,4-thiadiazole (TB), 2-amino-5-(2-hydroxyphenyl)-1,3,4-thiadiazole (TS), 2-amino-5-(2-hydroxy-5-sulfobenzoyl)-1,3,4-thiadiazole (TSF). In the fluorescence emission spectra (TS) of solutions with varying concentrations of hydrogen ions, a particularly interesting effect of dual fluorescence was observed. The aforementioned effect was observed even more clearly in the environment of butan-1-ol, relative to the compound’s concentration. Depending on the modification of the resorcylic substituent (TS and TSF), we observed the emergence of two separate, partially overlapping, fluorescence emission spectra or a single emission spectrum. Interpretation of the obtained spectra using stationary and time-resolved spectroscopy allowed the correlation of the effect’s emergence with the phenomenon of molecular aggregation (of a particular type) as well as, above all, the structure of the substituent system. The overlap of said effects most likely induces the processes related to the phenomenon of charge transfer (in TS) and is responsible for the observed fluorescence effects. Also, the position of the –OH group (in the resorcylic ring) is significant and can facilitate the charge transfer (CT). The determinations of the changes in the dipole moment and TD-DFT calculations further corroborate the above assumption. The following paper presents the analysis (the first for this particular group of analogues) of the fluorescence effects relative to the changes in the structure of the resorcylic group combined with pH effects. The results of biological studies also indicate the highest pharmacological potential of the analogue in the case where the effects of dual fluorescence emission are observed, which predisposes this particular group of fluorophores as effective fluorescence probes or potential pharmaceuticals with antimycotic properties.

## 1. Introduction

Contemporary medicine related to the treatment of conditions such as neoplastic, neurodegenerative, or mycotic diseases requires the application of scientific methods relying on the techniques of molecular spectroscopy and photophysics/photochemistry to provide an in-depth description of the physicochemical properties of new compounds offering the aforementioned properties. Therefore, both in the context of medicine and pharmacology, researchers employing the methods of molecular spectroscopy are faced with the very difficult and highly engaging task (also requiring specific specialist knowledge) of identifying new compounds displaying very particular molecular properties, oftentimes providing a multi-directional range of specific pharmacological effects. The selected molecules need to be very thoroughly analyzed in terms of their photophysical and photochemical properties to allow the identification of the analogues with the highest potential for actual medical applications.

**Scheme 1:**
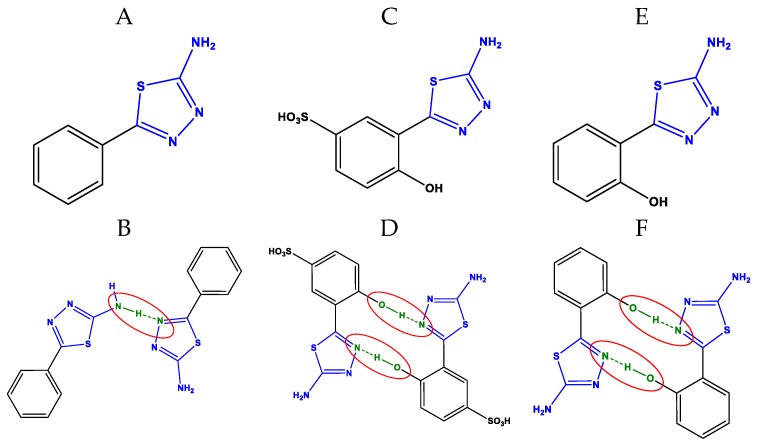
Chemical structure of compounds (**A**,**B**) benzoic thiadiazol (TB); (**C**,**D**) salicylic thiadiazol (TS); (**E**,**F**) sulfosalicylic thiadiazol (TSF) and their interaction between molecules (dimers).

Particular promise in the fight against the aforementioned diseases is shown by the compounds from the group of 1,3,4-thiadiazoles substituted with a resorcylic group (Scheme 1A–F).

1,3,4-Thiadiazoles are a group of new potential pharmaceuticals showing neuroprotective [1], anti-cancer [2], antibacterial [3], antioxidative [4], and antimycotic [3] properties. The following paper presents the results of spectroscopic analyses as well as theoretical and biological calculations conducted for three analogues from the 1,3,4-thiadiazole group.

For the performed study on the mechanisms of molecular interactions in aqueous solutions of varying pH as well as in selected organic solvents (of varying concentration), the following compounds were selected: 2-amino-5-phenyl-1,3,4-thiadiazole (TB), 2-amino-5-(2-hydroxyphenyl)-1,3,4-thiadiazole (TS), and 2-amino-5-(2-hydroxy-5-sulfobenzoyl)-1,3,4-thiadiazole (TSF) (Scheme 1A–F).

It is also noteworthy that the aforementioned 1,3,4-thiadiazole analogues show, apart from promising biological properties, also rather interesting spectroscopic properties. The same include the atypical effect of the tautomeric shift induced by changes in medium polarizability (changes of the keto/enol equilibrium [5]), effects related to crystal polymorphism [6] and solvatomorphism [7], changes in the fluidity dynamics of lipid membranes [8], or the effects of the emergence of various spectral forms (such as monomers/aggregates) in micellar systems [9]. Associating the aforementioned spectroscopic effects with changes of the photophysical/structural properties of said molecules can significantly contribute to our understanding of the phenomena underlying the pharmacological properties of the analyzed analogues.

However, the primary focus of the presented study was on spectroscopic analyses and quantum-mechanical calculations aimed at understanding the compounds’ unique fluorescence properties related to the effect of dual fluorescence emission. The analogues selected for the study (Scheme 1A–F) were analyzed in aqueous solutions with varying pH, supported by studies in selected organic solvents (for easier identification of aggregation effects and changes in the dipole moments between ground and excited states). The paper aims to explain the observed fluorescence effects related to the registered—in low pH conditions (as well as concentration dependent in solvents such as butan-1-ol)—effect of dual fluorescence TS or the emergence of several fluorescence bands (depending on the pH or the compound’s concentration). The study identified a correlation between the observed effects and a specific molecular form of the analyzed fluorophore for a given pH. The effects are particularly interesting as the selected analogues do not significantly vary in terms of the structure of their respective chromophoric systems. The most notable differences between the selected analogues are observed in the structure of their resorcylic substituent groups (Scheme 1A,C,E; not studied to date for this particular analogue group), which has not been subject to research in this type of media to date. By employing spectroscopic methods including electronic absorption and fluorescence spectroscopy, fluorescence lifetime and time-dependent density functional theory (TD-DFT) calculations, as well as calculations pertaining to dipole moment fluctuations between ground and excited states, we were able to demonstrate the complexity of the physicochemical processes responsible for the fluorescence effects (i.e., the emergence of dual fluorescence emission of several bands thereof). The conducted spectroscopic studies as well as quantum calculations and dipole moment determinations allowed us to correlate the observed fluoresce effects with the effects of aggregation taking place in the analyzed systems and influencing the capacity for a molecular charge transfer in said analogues. Furthermore, in order to confirm their potential medical applicability, the selected analogues were subjected to preliminary analyses of their antimycotic properties against a selection fungi species, as well as their antioxidative properties.

It is also noteworthy that in the literature [10], the phenomenon of dual fluorescence has been associated with the emergence of two separate emission spectra as a result of a single electronic excitation. The phenomenon can be induced in a variety of ways, including changes in the medium polarity, pH [11] or temperature [12], as well as aggregation effects. The theories attempting to explain the aforementioned fluorescence effects include:

- intermolecular charge transfer (CT) inducing the emergence of specific CT states [13], where the effect is combined with molecule twisting and twisted intramolecular charge transfer (TICT) occurs [14,15];
- emergence of the so-called excited-state intramolecular proton transfer (ESIPT) [16,17] in the molecules;
- in the case of additional concentration-induced effects, we are dealing with excimers [18];
- one should also mention processes related to breaking the Kasha rule (e.g., as recently described in detail by Brancato et al. [19]);
- a very interesting explanation of these types of effects provided in the literature also involves the processes related to molecular aggregation (i.e., aggregation induced emission (AIE)) [20,21].

However, it should be noted and emphasized that modeling the activity of fluorophores relative to the changes in the spectroscopic properties of the analyzed systems, both under conditions of varying pH and polarity, becomes particularly significant also in terms of designing specific fluorescence probes which are always in high demand in the context of molecular biology.

In the 1,3,4-thiadiazoles selected for the study, the emergence of fluorescence effects related to the dual nature of the same is also closely correlated with the structure of the substituent group used and modified (in our case for the first time) on the side of the resorcylic system (Scheme 1A,C,E). We observed that modification of this particular part of the substituent may significantly influence the aggregation properties (i.e., the way in which molecules aggregate) of the selected analogues. This observation was corroborated both experimentally and in the conducted quantum-mechanical calculations. Under specific circumstances (e.g., for TS in low pH medium or butan-1-ol), increasing the compound’s concentration may trigger processes related to a charge transfer between the interacting molecules. Studies conducted with the use of stationary and time-resolved fluorescence spectroscopy on the selected analogues from the 1,3,4-thiadiazole group allowed us to observe, in low pH media and butan-1-ol, that with increasing concentration, dual or multiple fluorescence bands emerged, partially overlapping (depending on the structure of the substituent group). Such effects were not observed for the analyzed group of compounds in pH higher than 5 or other solvents used in the study and significantly varying in terms of polarity. Furthermore, preliminary microbiological studies on the compounds’ antimycotic properties revealed an interesting correlation between stronger biological effects and the analogues displaying the more interesting/less typical fluorescence properties.

The primary goal of this work is to attempt to explain and give a brief description of the dual fluorescence effect observed in the selected analogues from 1,3,4-thiadiazoles including an aqueous environment with a different concentration of hydrogen ions, and their dependence on the structure of the substituent group on the resorcyl system side.

The presented paper marks out significant novelties in comparison to our previous work where selected analogues from 1,3,4-thiadiazoles were investigated mainly towards specific solvent effect and basic oxidative properties (thin-layer chromatography (TLC)-DPPH method with “dot-blot” test). In this paper, the authors intend to investigate if similar fluorescence effects will be observed in a more physiological environment and compare it with oxidative as well as more important antifungal activities of these molecules.

Uncovering the exact details of the changes in the molecular organization of the selected compounds in media with varying concentrations of hydrogen ions and varying polarity is significant in determining their potential for pharmacological and medical applications as well as the directions of further model studies in media such as micellar o liposomal systems. Such molecules may also be used as molecular probes very sensitive to environmental changes in systems studies in the context of molecular biology [22]. For this purpose, molecules such as 1,3,4-thiadiazoles which exhibit the dual fluorescence emission effect derivatives are in demand.

## 2. Results and Discussion

**Scheme 2:**
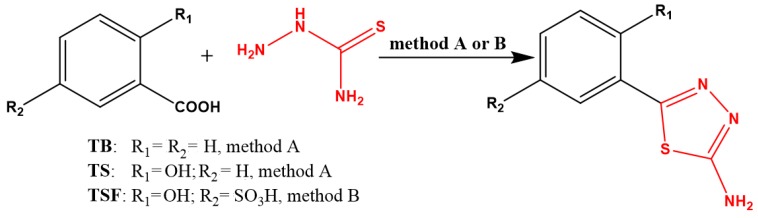
Synthetic pathway for the isolation of **TB**, **TS**, and **TSF**.

The selected compounds from the 1,3,4-thiadiazole group were synthesized in such a way so as to obtain significant differences in the structure of the substituent group in the resorcylic system (Scheme 1A,C,E; Scheme 2). In the case of 2-amino-5-phenyl-1,3,4-thiadiazole (TB), the structure consists solely of the benzene group bound directly to the 1,3,4-thiadiazole ring and an –NH_2_ group. 2-Amino-5-(2-hydroxyphenyl)-1,3,4-thiadiazole (TS) contains, in its benzene ring, a substituted hydroxylic group in the ortho position. In the case of 2-amino-5-(2-hydroxy-5-sulfobenzoyl)-1,3,4-thiadiazole (TSF), the benzene ring includes an –OH group substituted in the ortho position (analogically to TS). The remaining elements of the molecular structure are the same in all the analogues.

### 2.1. Analysis of Spectroscopic Effects

**Figure 1:**
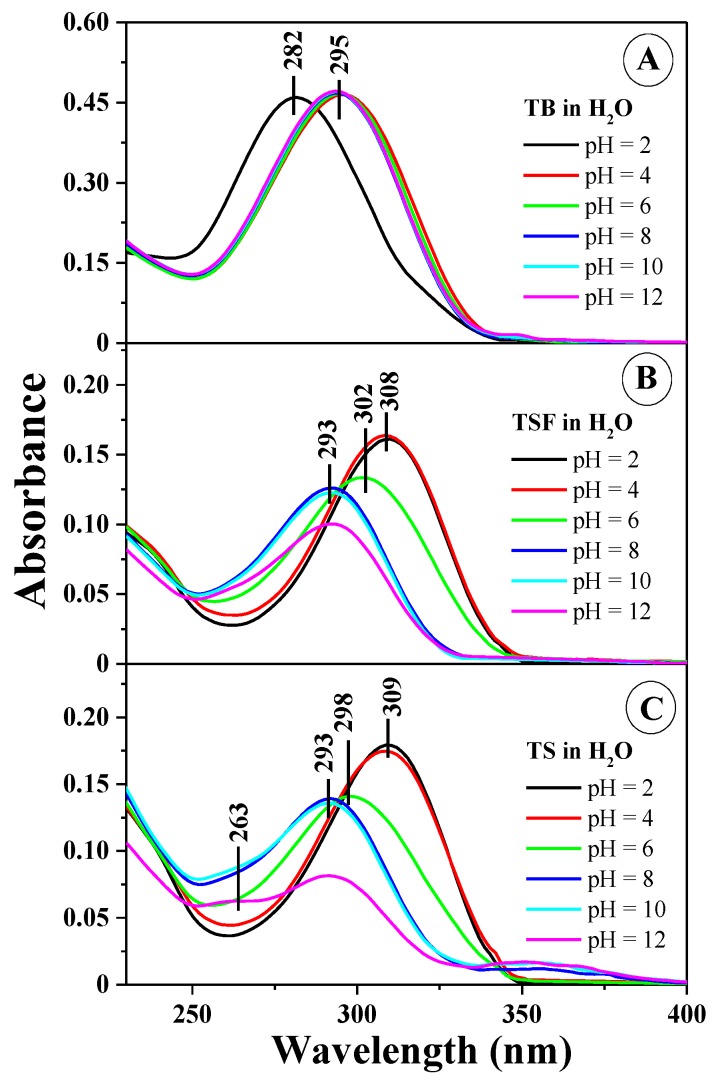
Absorption spectra of thiadiazoles TB, TSF and TS dissolved in H_2_O at varying pH (from 2 to 12). (**A**) TB; (**B**) TSF; and (**C**) TS. The spectra were not normalized. The absorbance intensity was adjusted to avoid the reabsorption effects.

Figure 1 presents the electronic absorption spectra for the 1,3,4-thiadiazoles selected for the study, at varying concentrations of hydrogen ions in the aqueous solution used, respectively for TB in Figure 1A, TSF in Figure 1B, and TS in Figure 1C (for clarity of the presentation, spectra for pH 2, 4, 6, 8, 10, and 12 were selected).

The obtained results indicate very clear changes in the shape of the spectra, particularly in the region significant to physiological values. For the solvents used in the study, the positions of the absorption and fluorescence maxima as well as the respective Stokes shifts are presented in Table S1 (see the Supplementary Materials). As clearly visible in Figure 1B,C, the dissociation of the –OH group from the resorcylic ring in the ortho position (Scheme 1C,E) results in a clear hypsochromic spectral shift by 301 cm^−1^ for TSF and 303 cm^−1^ for TS in the case of spectra observed at pH 12, and bathochromic shift by 1016 cm^−1^ for TSF and 684 cm^−1^ for TS in the case of spectra observed at pH 2 (as well as pH 1) relative to the spectra of the same compounds at pH 7. For TB, Figure 1A reveals only a slight shift of the bands within the pH range of 6–12 (without the –OH group in the resorcylic system). As evident in the case of all analyzed analogues, the process of ionization may also be accompanied by the processes of compound aggregation [23,24]. At pH of approximately 6–8 (depending on the substituent), a very clear broadening of the absorption spectrum can be observed (mainly) for TS and TSF (the effects are significantly less prominent for TB) (Figure 1A), indicating the possible presence of forms other than the monomeric spectral forms of the analyzed structures [9]. In the case of TS and TSF spectra at pH ~2, the spectral absorption is the least pronounced, which may suggest a significant prevalence of aggregated forms in the case of these analogues (see Figure 1B,C). Meanwhile, no such drastic decrease of the absorbance level was observed for TB within the same range and it was significantly higher at the same pH (compared to TS and TSF). This also clearly evidences the influence of the structure of compounds selected for the study on the manner in which aggregated molecular structures are formed. In the case of TB at pH 2, the compound’s absorbance was slightly decreased relative to other spectra recorded for higher pH, suggesting an increase in the number of the analogue’s monomeric forms. In the case of TS and TSF, such behavior was not observed in the studied concentration range, which may confirm the fact that the molecules of those analogues interact more strongly and are therefore able to form more durable aggregated forms (Scheme 1B,D,E). It is also noteworthy that in the case of TB at pH 2, the spectrum shifts (primarily) towards the shorter wavelengths, which, according to Kasha’s exciton splitting theory, may suggest the presence of card pack analogues (the most likely for this analogue). Moreover, for low pH values it cannot be excluded that the thiadiazole ring is subject to protonation and an ionic form with the -N^+^-H group is formed in all the studied analogues. The effect was most evident in the case of TB but was also present for TS and TSF, although the latter two analogues containing an –OH group may additionally be subject to head to tail aggregation as well. This was reflected in the absorption spectra where the long wavelength spectra registered for this analogue revealed a clear broadening of the bands. In the case of TSF, we observed what most likely was a greater equilibrium between the respective types of aggregation together with molecule ionization, whereas for TS, we recorded a band with the maximum at approx. 350 nm, evidencing the possible presence of aggregated structures even larger than dimers.

Moreover, in the case of TS a (fairly) clear isosbestic point could be observed at approx. 345 nm, which may suggest that the transfer between the respective aggregation forms in the compound is highly fluid (and the molecules are susceptible to such interactions). For the band located in the long wavelength side, this points to a stronger aggregation (head to tail) whose level is most likely constant at a low pH. The intensity of bands evidencing the aggregation effects occurring in the selected analogues is also significantly correlated with the structure of the very substituent group in the resorcylic ring bound to the 1,3,4-thiadiazole ring.

**Figure 2:**
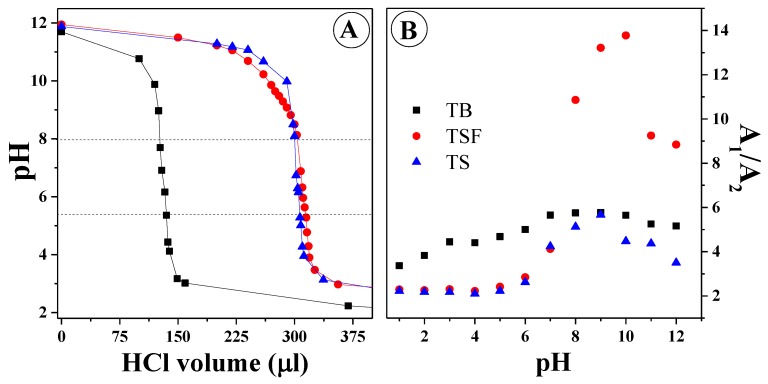
(**A**) pH-metric titration curves for TB (black squares), TSF (red circles), and TS (blue triangles). Titration was carried out with the use of 1 M HCl. (**B**) The ratio of the maximum electron spectra at c. 309/330 nm for TB, TSF, and TS relative to changes in the pH of the aqueous solution.

Figure 2A presents the titration pH-metric curves and pK points for the ionization-related –OH groups in the resorcylic ring of TS and TB, as well as the pK points for all compounds for the -N^+^-H group.

In the case of TS and TSF, the pK for the –O^−^ group was, respectively, 7.35 and 7.02, and for the –N^+^-H group, the pK for all the compounds was approx. 4.85. For pK higher than the larger of the above, we observed for the respective analogues (TS and TSF) the prevalence of forms with the –OH group, whereas for pK below the lower of the above, for each analogue the prevalence of forms with the –N^+^-H group was observed. Figure 2B presents the ratio of absorbance intensity at approx. 309–330 nm in all compounds relative to changes in the pH of the aqueous solution. It can be observed that for pH from 1 to approx. 7, TS and TSF clearly showed a drastic transition between the forms with the aforementioned groups subject to ionization (-O^−^ and –N^+^-H). The most significant change was observed for TSF (for which we could probably observe the greatest equilibrium/transfer between the respective types of ionized forms that could undergo the given types of aggregation) and the changes were also relatively clearly manifested for TS. In the case of TB, the changes of this type were the least extensive, as confirmed by the observations in Figure 1A. The significant reduction of this ratio in the case of TS and TSF further confirms the occurrence of the aggregation effects discussed above. It also corroborates the conclusion that the process of nitrogen atom protonation was in equilibrium and did not affect the intensity of the ratio for the selected 1,3,4-thiadiazole analogues within the pH range of 1 to approx. 5/6.

**Figure 3:**
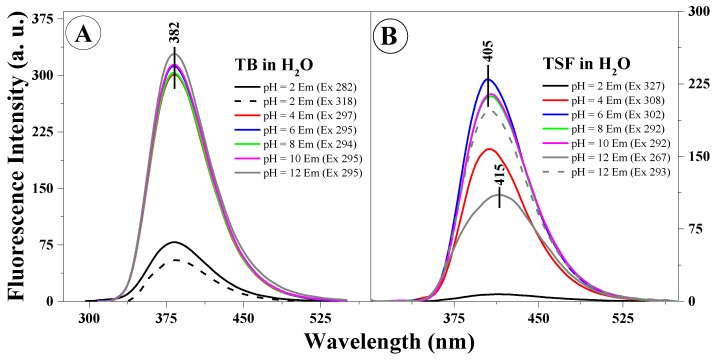
Fluorescence emission spectra of (**A**) TB and (**B**) TSF dissolved in H_2_O at different pH. The excitation was set at the absorbance maximum of each sample, respectively, as pointed out in the figure. The spectra were measured at room temperature.

Figure 3A,B presents example fluorescence emission spectra for TB (Figure 3A) and TSF (Figure 3B) corresponding to the respective absorption spectra.

The excitation wavelength for all the analyzed samples corresponds to the absorption spectra maximum (Figure 1A,B). In the case of the fluorescence emission spectra in TB, we observed virtually no shifts relative to changes in the solution pH or excitation wavelength (at the maximum characteristic of monomeric or aggregated forms), only significant, pH-dependent changes in intensity (the intensity of fluorescence decreased with decreasing solution pH). In Figure 3B, presenting the fluorescence emission spectra for TSF, we observed both a slight shift of the fluorescence emission bands correlating to the pH of the analyzed solution, and changes in emission intensity as well as a noticeable change in the band shape depending on the excitation wavelength, which confirms the possible presence of both respective ionized forms and specific spectral forms (i.e., monomeric or aggregated forms or for example, dimers). We can therefore clearly observe the influence on the location of the emission band maximum for the given compound relative to the structure of the substituent group. However, in all the presented fluorescence emission spectra, a single fluorescence band was observed with the maximum at approx. 382 nm (TB) or 405 nm and approx. 415 nm (for TSF). It is noteworthy that in the case of the selected analogues, changing the concentration of hydrogen ions in the medium has a significant impact on the stability of the excited state of the given molecule in the particular solvent. It is noteworthy that in the case of selected analogues TB and TSF the emission observed is predominant in their monomeric forms.

**Figure 4:**
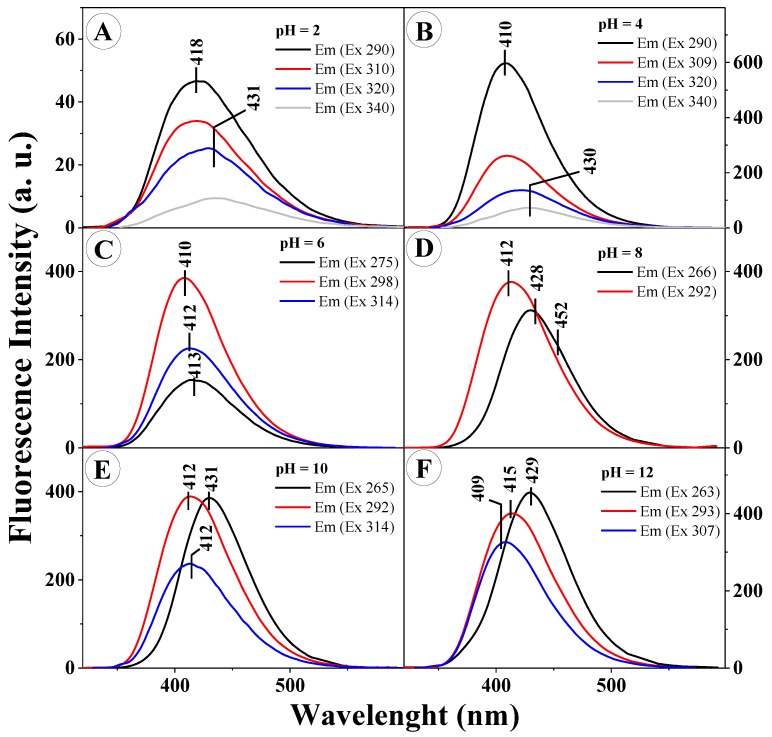
Fluorescence emission spectra of **TS** dissolved in H_2_O at different pH: (**A**) pH = 2, (**B**) pH = 4, (**C**) pH = 6, (**D**) pH = 8, (**E**) pH = 10, and (**F**) pH = 12. The excitation was set at the absorbance maximum of each sample, respectively, as pointed out in the figure. The spectra were measured at room temperature.

However, the most interesting effects observed in the presented study were those presented in Figure 4A–F.

In order to provide a further, more in-depth insight into the character of the molecular interactions occurring for TS in aqueous solutions of varying pH (at pH levels corresponding to the results in Figure 1C), its respective emission spectra were presented for various excitation values. As can be clearly noticed, for different excitation wavelengths (corresponding to the absorption spectra in Figure 1) we observed the emergence of several fluorescence emission bands, shifted relative to each other, or the effect of dual fluorescence in the case of pH 2 and 4 (grey line in Figure 4A,B) for the excitation wavelength corresponding to the aggregated form, such as a dimer (in the head to tail layout). For all the presented spectra, the excitation wavelength used corresponded to the prevalence of the monomeric form (short-wave excitation at the wavelength of approx. 290 nm) and the prevalence of the aggregated form (excitation in the long wavelength side of the spectrum). Figure 4A,B presents the emission spectra for TS at the pH of 2 and 4, respectively. Noticeably, after excitation with the wavelength corresponding to the maximum of the emission spectrum and the maximum characteristic of aggregated forms, the emission spectrum produced a clearly visible dual fluorescence effect (grey line). For pH higher than 5, depending on the length of the excitation wave, we observed clear shifts of the fluorescence emission spectra but with only single emission bands. It should be pointed out, however, that said bands overlapped to a considerable extent and their emission maxima corresponded to the first or second emission band from Figure 4A,B. One should also mention the very clearly visible correlation between the varying locations of the emission bands and the excitation wavelengths applied. Depending on the excitation wavelength used (on the short wavelength side approx. 290 nm or on the long wavelength side approx. 320 nm) aggregated forms of a specific type were more commonly excited, respectively card pack or head to tail forms (as further confirmed in the TD-DFT calculations). Naturally, the emergence of the respective aggregation forms was, in this case, closely related to the particular solution pH, which will be further discussed in the following part of this paper. The technique of resonance light scattering (RLS) allows a highly probable identification of the effects related to the given type of chromophoric aggregation of the studied analogues [25], as well as the differing character of such aggregation interactions.

Based on the exciton splitting theory and by employing quantum-mechanical TD-DFT calculations, we were able to conclude that the emergence of the very interesting and atypical spectral shifts in the emission spectra registered for TSF and, most notably, the effect of dual fluorescence observed for TS in the pH range of 1–4, was significantly influenced by the presence of different aggregation forms, specifically card pack aggregation (more characteristic for short wavelength excitation) and head to tail aggregation (characteristic for long wavelength excitation) of the analyzed molecules. Furthermore, by using the spectral shift in the absorption spectra in combination with the mechanism provided by the exciton splitting theory, we were also able to calculate the distances between adjacent chromophores of the analyzed molecules [23,26]. So, for the potential TB dimers, the distance between adjacent chromophores obtained in the calculations was approx. ~3.67 Å, whereas for TSF that distance was approx. ~3.61 Å [26]. In the case of TS, the distance was the smallest at approx. ~3.52 Å. Thus, in the case of TS and TSF, aggregational interactions very strongly influence the observed fluorescence effects.

To recapitulate at this point, one should strongly emphasize the impact on the observed spectral effects (in particular in the emission spectra of TS and TSF) of such factors as, above all, the phenomenon of molecular aggregation (of two types) and difference in the substituent structure of the respective compounds (which naturally partially correlates with the former of the two factors).

We can therefore preliminarily posit that the fluorescence emission spectra of the analyzed compounds under high pH conditions are dominated by the emission from monomeric forms; various ionized forms, and at low pH, in the case of TS/TSF, form the two aforementioned aggregated forms. For TS, at pH lower than 5, the aggregation processes may, given the prevalence of card pack aggregative interactions, also trigger effects related to intermolecular charge transfer (CT), which influence the dual character of emission spectra, as confirmed in the conducted TD-DFT calculations as well as, most importantly, calculations of the dipole moment in the ground and excited state (described below).

Next, Figure S1A,B presents the fluorescence excitation spectra (Ex) for TB (Figure S1A) and TSF (Figure S1B), corresponding to the fluorescence emission spectra in Figure 3A,B.

Emission in the case of excitation was registered at the wavelengths corresponding to the maxima of the respective fluorescence emission spectra in Figure 3A,B. The much higher selectivity of the fluorescence excitation spectra compared to the electronic absorption spectra facilitates the excitation of a specific molecular form or compound in the analyzed analogue. In other words, the fluorescence excitation spectra allow the excitation of a specific spectral form of the compound (e.g., the monomer, dimer, etc.). Figure S1A presents the fluorescence excitation spectra for TB at the selected pH levels (corresponding to the results discussed above). It can be observed that in the case of TB, virtually only the band characteristic for the compound’s monomeric forms was observed (varying in intensity depending on the pH). However, said bands were quite significantly widened, which suggests that even in the case of this analogue the effects of aggregative interactions may be observed. Figure S1B for TSF presents, apart from the band with the maximum at approx. 300 nm, a very clearly visible band on the long wavelength side with the maximum at approx. 360 nm, varying in intensity depending on the excitation wavelength (short or long wave excitation, as described above) and pH, characteristic for aggregated forms emerging in correlation with the presence of this particular ionic form of the compound. So, in the case of TSF we observed the highest intensity of fluorescence excitation bands in the region associated with aggregation (head to tail, long wave). As already mentioned, the fluorescence emission spectra for TSF revealed, in correlation with the solution pH, bands that were partially overlapping and shifted relative to each other.

For TS, Figure S2 presents excitation spectra analogical to those observed in TB and TSF and presented in Figure S1, at varying pH levels of the aqueous solution (analogically to the emission spectra in Figure 4). Similarly, to TSF, in the case of TS we observed, depending on the pH and excitation wavelength, band enhancement on the long wave side (which evidences the prevalence of card pack aggregation). As visible in the excitation spectra, bands originating from the aggregated forms are more strongly evidenced for pH levels at which the emissions spectra showed effects related to dual fluorescence, i.e. pH 2 or 4.

Within these pH ranges, the spectra show a significantly increased half-value width relative to other pH levels, which clearly suggests the presence of other than monomeric forms of the compound. Thus, for TS (and to a considerable extent also TSF) we can observe the strongest effects related to exciton splitting at the main energetic level S_0_, which induces the respective changes in the fluorescence emission spectra. In the pH ranges where the effects of dual fluorescence or multiple bands significantly shifted relative to each other are not observed, the excitation spectra at wavelengths corresponding to the respective forms of the analyzed compounds are fairly consistently overlapping within practically the entire wavelength range.

**Figure 5:**
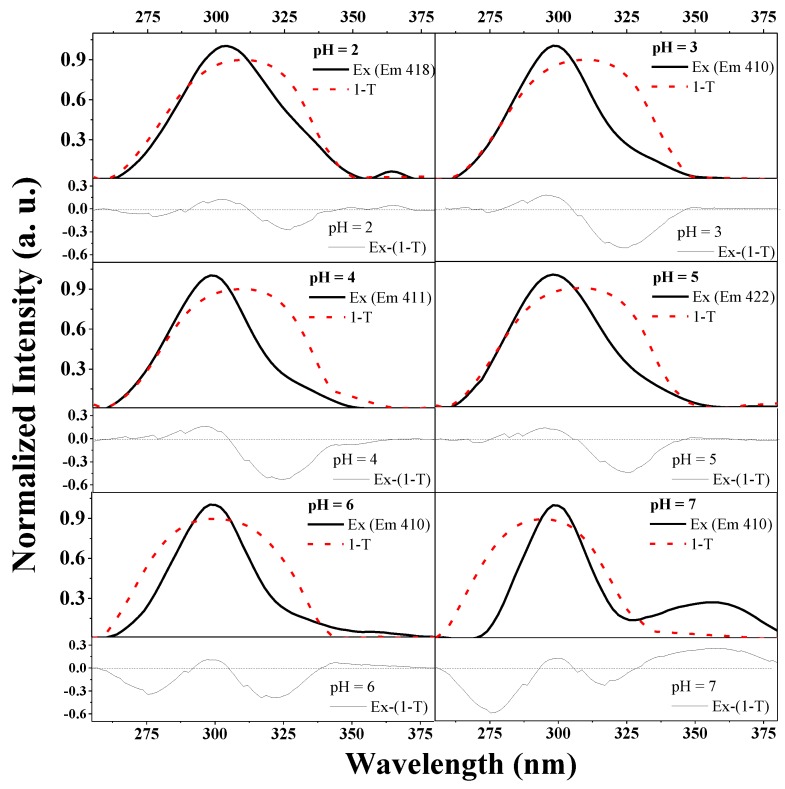
Fluorescence excitation spectra normalized at the maximum (to the value of 1) in comparison with 1-T spectra (T: transmission) of TS dissolved in H_2_O at different pH (from 2 to 7). Below, the respective differential spectra Ex-(1-T) corresponds to the spectra in the panels above.

To emphasize the influence of the type of aggregation on the observed fluorescence effects, one needs only to carefully analyze the results presented in Figure 5.

The figure illustrates the overlap between the normalized excitation spectra (presented in Figure S2 for TS) and the 1-T spectra for this compound (T: transmission) and the differential spectra under each respective panel (Ex-(1-T)). Such a presentation of the spectra allows us to observe the impact that a certain type of aggregation has on the aforementioned fluorescence effects. For the pH levels at which the effect of dual fluorescence is present, we observed band enhancement (in the differential spectra) on the long wave side (characteristic of head to tail aggregation) (e.g., for pH 2–5). Whereas, with increasing pH (at 6 two types of aggregation are evident and the enhancement is present on both sides), already at the level of pH 6, we observed increased enhancement on the short wave side, which implies the prevalence of head to tail aggregation, and in the emission spectra, the disappearance of effects related to dual fluorescence emission.

**Figure 6:**
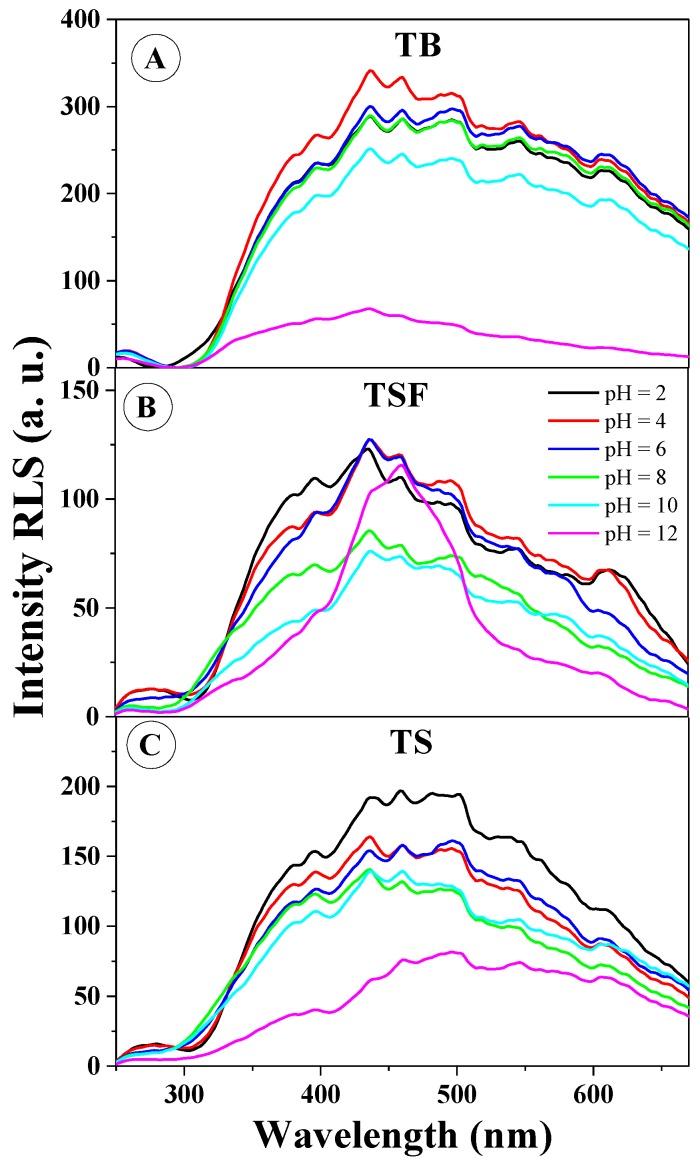
Resonance light scattering spectra (RLS) of (**A**) TB, (**B**) TSF, and (**C**) TS dissolved in H_2_O in different pH.

To recapitulate, based on the theory of exciton splitting, the characteristic long wave bands should, in the case of the analyzed analogues, be associated with various types of aggregated forms, as further confirmed by the RLS spectra (presented in Figure 6 and Figure SA3).

According to the exciton splitting theory, the observed shifts are related to the prevalence of card pack aggregation, long wave enhancement, and in TB with head to tail aggregation [23]. Based on the above, it can be inferred that the observed fluorescence effects are certainly related to the effect of molecular aggregation taking place in the used solutions of varying pH. In the case of TS, the card pack aggregation facilitates the emergence of intermolecular CT states (as confirmed by dipole moment calculations discussed later in the text), whereas in TSF molecules, due to the structure of its substituent group, despite the most likely very heavy card pack aggregation the preference is also for head to tail aggregative interactions and a charge shift evidenced by the split of the fluorescence emission spectra into two bands characteristic of the respective electronic states.

As the subsequent research step, in order to confirm one of the main (and necessary) factors influencing the effects observed in the fluorescence spectra (i.e., aggregation), spectral RLS measurements were conducted (∆λ = 0) for the selected derivatives in the entire pH range. Figure 6 presents the RLS spectra for TB (Figure 6A), TSF (Figure 6B), and TS (Figure 6C). As provided in the literature, the occurrence of RLS spectra should usually be associated with chromophoric aggregation of the interacting molecular systems [25]. It is clearly apparent that the RLS spectra confirm the above assumption related to the significant impact of aggregation effects on the changes in fluorescence spectra observed above. Figure 6A illustrates the presence of RLS bands for TB in practically the entire pH range; however, the spectra (except only high pH in the range of 10–12) only slightly differ from each other, similarly to fluorescence emission spectra for this compound relative to the changing pH. Figure 6B presents the RLS spectra for TSF at selected pH levels; the tendency here is very similar to that observed for TB, however, in this case the shifts recorded in the emission spectra are considerably more significant (and the shape of RLS spectra for TSF is considerably different than in the case of TB, which suggests the presence of different aggregation forms). In Figure 6C, similarly to the other analogues (presented in Figure 6A,B), RLS spectra are also clearly present in practically the entire range of hydrogen ion concentrations (slightly less prominent at high pH). One should emphasize at this point that RLS spectra were observed for all the analyzed analogues, irrespective of the changes observed previously in the emission fluorescence spectra. Within the wavelength range of 350 to 420 nm, we registered the most significant changes in RLS scattering for the studied analogues, which corroborates the significant participation of head to tail aggregation in the observed aggregative effects, as already discussed in the context of absorption and fluorescence excitation spectra. The effects are very evident in Figure S3, representing the ratio of RLS scattering intensity at the wavelength of approx. 436 nm relative to pH, where one can directly observe that RLS spectra are present practically in the entire range of pH levels.

In summary, it is noteworthy at this point that the RLS spectra related to chromophoric aggregation of the analyzed molecules are very characteristic and clearly correlate the observed fluorescence effects with various types of said aggregation. However, it should also be emphasized that the same is not the only factor determining the observed fluorescence effects as very intensive RLS bands were also observed for TB where, as discussed in the context of previous experiments, we observed no significant changes in the fluorescence emission spectra relative to the emergence of dual fluorescence or presence of multiple fluorescence emission bands partially shifted relative to each other. The oscillative structure of the observed RLS bands evidences the abundance of aggregated structures of varying sizes in the analyzed compounds [25] and may confirm our earlier assumptions regarding the presence of different aggregated forms of the analyzed molecules. In the pH range in which a greater aggregation tendency of molecules is observed, the effects of broadening the emission band or its significant shift or other dual fluorescence emissions-related effects are observed much faster. Both the excitation and RLS spectra explicitly relate these two factors together. The aggregation process with appropriate molecular structure (and above all the application of its conformation) can cause changes in fluorescence emission spectra.

**Figure 7:**
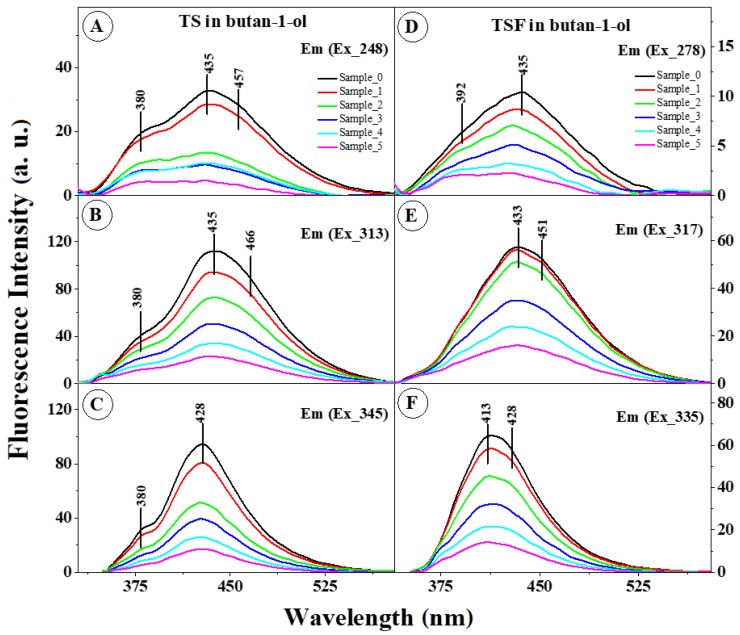
Fluorescence emission spectra of TS and TSF dissolved in butan-1-ol. For TS: (**A**) excitation at 284 nm; (**B**) excitation at 313 nm; and (**C**) excitation at 345 nm. For TSF: (**D**) excitation at 278 nm; (**E**) excitation at 317 nm; and (**F**) excitation at 335 nm. Measurements were carried out for six different dilutions: sample 0: 3 mg of TS/TSF dissolved in 2 mL of butan-1-ol; sample 1: sample 0 + 1 mL of butan-1-ol; sample 2: sample 1 abstract 1 mL + 1 mL of butan-1-ol; sample 3: sample 2 abstract 1 mL + 1 mL of butan-1-ol; sample 4: sample 3 abstract 1 mL + 1 mL of butan-1-ol; sample 5: sample 4 abstract 1 mL + 1 mL of butan-1-ol.

Nonetheless, it can be concluded with considerable certainty that in the case of the observed fluorescence effects, aggregation is a factor necessary for their emergence. The above hypothesis is corroborated by the results presented in Figure 7A–F as well as in Figure S4A,B.

Figure 7A–C presents the fluorescence emission spectra for TS at three different excitation wavelengths (248 nm, 313 nm, and 345 nm, respectively) relative to the concentration of the analyzed compound. It can be observed that regardless of the length of the excitation wave, with increasing compound concentration the emission also grows significantly at approx. 435 nm in the dual fluorescence spectrum. This clearly evidences the impact of aggregation effects on the studied fluorescence effects. An analogical experiment was conducted for the TSF derivative (i.e., one involving three excitation wavelengths and variable compound concentrations). We observed, firstly, that the bands with the maximum at 413 nm (Figure 7F) and 433 nm (Figure 7E) showed a significant loss in intensity with the increasing concentration but nonetheless remained present. Secondly, even for TSF (where spectra measured for varying pH did not show this effect) we observed a clear effect of dual fluorescence in Figure 7D under short wave excitation (e.g., sample 5). Furthermore, it is important to notice that more intensive short-wave emission was observed under short wave excitation in the region characteristic of head to tail aggregation enhancing the card pack aggregation, which is clearly visible in Figure S4 (black dots on both panels).

In order to better understand the mechanism of the spectral shifts described above (Figure 1, Figure 3, Figure 4) as well as the fluorescence effects observed in the compounds, as shown in Figure S5 and Figure 6A, we presented the correlation between Stokes electronic transition π→π* and the changes in the function of polarity fluctuations $E_{T}^{N}$ as well as *F*(*ε*,*n*) which describe changes in the polarity/polarizability of the solvent used.

Firstly, as can be observed for all selected compounds (Figure S5, Figure S5A), the slopes of the lines are positive for all the solvents used, which usually signifies an increase in the dipole moment of the excited molecule and may suggest that its direction is preserved during the electron transition. This fairly clearly evidences the possibility of CT between the molecules triggered by aggregation interactions, causing the particular observed fluorescence effects, such as dual emission in the case of TS. Furthermore, it confirms the significant impact of aggregation effects on the non-specific interactions taking place between the molecules of the analyzed compounds. Furthermore, the somewhat irregular linear dependencies observed in the presented relationships (Figure S6B) largely confirm the possibility of triggering aggregative interactions by hydrogen bonds, particularly in environments where dual fluorescence emission or two separate, largely overlapping, emission bands are observed. The presence of solvent molecules modifies the absorption and fluorescence spectra of the analyzed compounds, usually leading to shifts in the locations of their maxima towards longer, less frequently shorter wavelengths. However, the presence of hydrogen bonds (triggering aggregation which may influence CT-related effects) may lead to significant departures from that model [27]. Furthermore, one should also consider the fact that in systems such as these studied here, we observe a continuous equilibrium between the formation of intermolecular and intramolecular hydrogen bonds. The former lead to the formation of aggregated systems (firstly of the dimer type), as clearly evidenced by the changing intensity of RLS spectra and fluorescence excitation spectra.

Most notably, however, one should take into account the results of dipole moment calculations performed for the analyzed molecules in ground and excited states based on their respective absorption and fluorescence emission spectra in correlation with Reichardt’s [28] and Kawski’s methods [29], respectively, Equation (2) and Equations (5)–(7). The most important factors pertinent to those calculations are presented in Table 1 for the former and Table 2 for the latter method, for comparison and corroboration of the results.

As we can see, the most significant changes of the dipole moment of the analyzed molecules (between the ground and excited states) were observed for TS molecules and TSF, the compounds whose fluorescence emission spectra revealed the most interesting fluorescence effects (depending on the excitation, either two largely overlapping fluorescence emission bands or dual fluorescence). The above calculations confirmed in this case that CT occurred between the analyzed molecules. In the case of TB and TS we observed the ratio in the dipole moment between the excited and ground states of 1.50 whereas in the case of TSF the difference increased to 1.62 (D). Results of the above calculations confirm the significant influence of the structure of the substituent system on the observed fluorescence effects. They also suggest that an important effect of solvatochromic shifts entails the intersection of states with varying distributions of electronic density dictated by the polarity of the respective medium. In polar solvents, electronic states with high dipole moments (often in fact the CT states) are stabilized relative to the states with low dipole moments. If such states are characterized by similar energies, a change in the polarity/polarizability of the medium may lead to a reversal of the states’ order. Since photochemical properties are determined by the character of the lowest excited state of the given multiplicity (Kasha’s rule), they can be (as observed in the present study) subject to very significant changes with the solvent’s polarity.

### 2.2. Fluorescence Lifetime Analysis

In the subsequent part of the study on the mechanism of molecular interactions inducing the respective fluorescence effects in the emission spectra, as presented above in Table S3 (and Figure S7), we considered the results of time-resolved measurements of fluorescence lifetimes conducted for all analyzed analogues in the full range of pH levels.

The methodology is presented and described in detail in Section 3 (Section 3.5). Fluorescence decay for TSF was single-exponential. The measured fluorescence lifetime was between 0.37 and 5.43 ns, depending on the pH of the solvent (the correlation is presented in Figure S7). A clearly visible reduction of the fluorescence lifetime was observed for pH < 4.

The correlation between fluorescence decay and pH observed for TS is similar to that obtained for TSF. For pH <4, we recorded a significant reduction of the fluorescence lifetime. Moreover, for this analogue we observed two exponential fluorescence decay at pH = 2, which suggests the possible presence of more than one molecular form in the solution. The measured fluorescence lifetime for the main component, 1.64 ns, fits in well with the observed decrease of the parameter at low pH levels, with the share of this component estimated at 72%. The fluorescence lifetime recorded for the second component was considerably shorter (0.29 ns). At pH within the range of 4–12, the fluorescence lifetime for the TS analogue was decisively shorter than in the case of TSF but similarly to the latter, it remained at a roughly constant level.

In the entire range of pH values, the fluorescence lifetime recorded for TB was the shortest among all three analogues (0.1–0.18). In the case of this analogue, the second constituent, with the fluorescence decay time of 1.2 ns and relative share in total fluorescence estimated as 11%–20%, was observed both at low (pH = 2) and high (pH = 11,12) pH levels. Clearly, aggregation which triggers the effects associated with dual fluorescence takes place in TS and TSF in practically the whole range of pH values (the lifetime remains at a fairly constant level). For TB, at pH lower than 7, we observed a far more prominent presence of the second constituent in the overall lifetime, which indicates the existence of spectral forms other than aggregates. The reasonability of using fluorescence lifetime measurements in this paper is worth emphasizing. In particular, due to the process related CT, a significant increase in the average fluorescence lifetime is observed. Hence, the effect of dual fluorescence reported in this work, allowed for the discovery of noticeable changes in the fluorescence lifetime of the fluorophore, which together with noticeable aggregation processes, fully confirms our hypotheses.

To recapitulate the deliberations so far, it should be emphasized that (as largely corroborated by the results of quantum-mechanical calculations) effects observed in the case of TS and TSF molecules (in high concentrations) are triggered primarily by the intermolecular phenomenon of charge transfer (CT) enforced (or facilitated) in this particular case by the phenomenon of molecular aggregation of a given type (e.g., head to tail or card pack). Also, the structure of the substituent group on the side of the resorcylic system significantly influences the described overlap of two phenomena.

**Figure 8:**
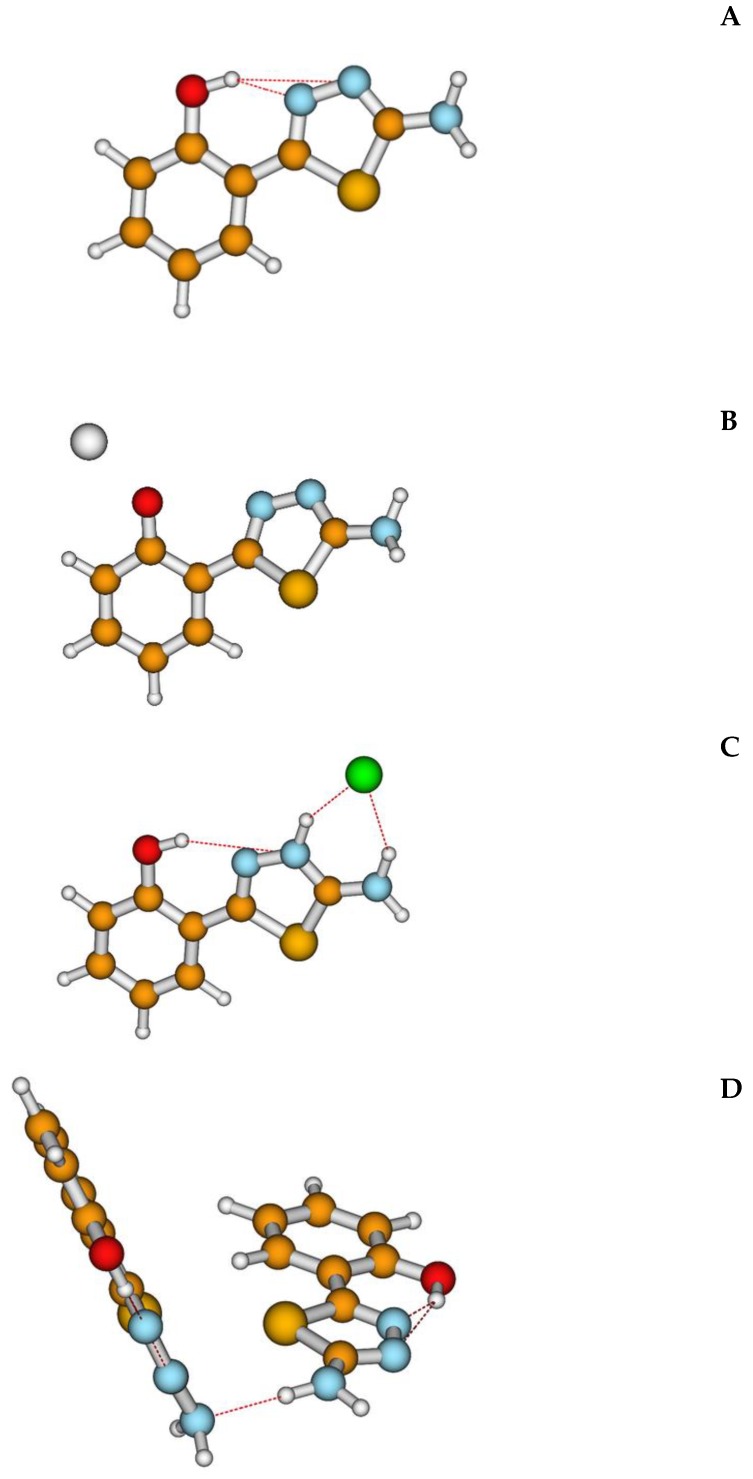
Ground state structures of TS^−^ (**A**), TSH (**B**), TSH^2+^ (**C**) and (TSH)_2_ (**D**) as optimized using B3LYP/aug-cc-pVDZ and polarizable continuum model (PCM) with water as the solvent.

Figure 8 (and Table 3) provides a schematic, structural presentation of the TS^−^ molecule, TSH molecule, TSH^2+^ and sample TS dimer as optimized using B3LYP/aug-cc-pVDZ and polarizable continuum model (PCM) with water used as the solvent. Table 4 summaries the main predictions regarding the excited state energetics of these species.

The main computational findings are as follows. Firstly, the TS molecule in the form of TS^−^, TSH, and TSH^2+^ has a single, low-lying and intensive excited state responsible for the shape of its absorption and emission spectra (Table 3, Figure 8A–C). However, in the case of the TSH molecule, the situation changes drastically. The TS molecule may form a hydrogen bond both within its own structure, which in itself affects its spectroscopic properties, as well as with the molecules of the medium. Hence the single intensive TS state is split in two in this case. This results in a more complex, dual structure of the TS absorption spectra, as observed in Figure 1, where the absorption spectra reveal band widening on the long wave side. The subsequent analyzed TS dimer, (TSH)_2_ is only one of many potentially possible types of aggregates (as already discussed and described above in the context of the experiments), therefore the results should be treated as only semi-quantitative (although significant as a theoretical confirmation of the experimental results). Nonetheless, the calculations offer a certain insight into the experimentally analyzed photophysics. As shown in Figure 8, the structure of the TS dimer includes an intermolecular hydrogen bond, apart from the possible intramolecular hydrogen bonds characteristic of monomers. The interaction between monomers splits the two excited states of the TS molecule into four dimer states which transfer intensity onto the respective bands present in the fluorescence emission spectra (Figure 3 and Figure 4). This fact has a significant bearing on the absorption spectra and may in fact be key to the overall fluorescence behavior. In one of the discussed dimeric states the energy of vertical emission is very strongly shifted towards red (bolded in Table 3), which may account for the observed dual fluorescence; the high energy band is monomeric whereas the low energy band is dimeric.

Also, it is worth mentioning that the dual fluorescence observed in the selected 1,3,4-thiadiazole analogues may occur via a pathway other than that proposed in this work (two independent modes of action may be possible). Based on the DFT calculations, the investigated compounds in their ground state occur solely as enol tautomers. Excitation of the enol form would likely enable an additional and independent process resulting in dual fluorescence emission. Once in the excited state either cis or trans-enol the enol form may tautomerize to the excited keto-form, which in turn may emit the long wavelength fluorescence.

Since the high concentrations are characteristic of aggregation-related dual fluorescence effects, this second and independent process may be dominant at low concentrations. The possibility of such mechanism is justified by the lack of clear differences between absorption spectra of the investigated derivatives recorded in series of various organic solvents. Moreover, according to our preliminary calculations, the energy differences between the excited enol and excited keto form are relatively small, which is consistent with the proposed relaxation pathway.

In a conclusion, the hypothetical second mechanism is proposed on the basis of preliminary calculations and relies upon an excitation of the neutral TS molecule, which in its ground state occurs as enol tautomer. The excitation of the enolic TS results either in straightforward fluorescence emission from the excited enol tautomer (higher energy emission band) or the excited keto tautomer is formed as result of proton (hydrogen) transfer from the phenolic –OH to the nearest thiadiazole nitrogen atom. The excited state of such keto tautomer is energetically similar to that of the excited enol and may emit photons manifesting as the second (lower energy) emission band, with subsequent return to the enolic form characteristic of the ground state.

The proton transfer into one of the nitrogen atoms is favored by the conformation in which the phenolic –OH resides near the N rather than S atom of the thiadiazole ring. Based on crystal structures reported to date [7], such conformation is highly likely.

### 2.3. Antioxidative Properties of the Analyzed Compounds

The determination of antioxidative potential was performed with a standard spectrophotometric test with the use of a solution of DPPH^•^ radicals [30,31,32]. DPPH is a relatively stable radical which, when dissolved in alcohol, produces a solution with a strong violet color with the absorption maximum at λ_max_ 519 nm. In the presence of a compound with potential antioxidative properties, it undergoes reduction which results in the decay of the absorption maximum at λ_max_ 519 nm and color change from violet to yellow. The changes are monitored spectrophotometrically. The antioxidative capacity of a given compound is determined by the speed at which the given analyte solution of known concentration reduces the fixed concentration of the radical solution. The obtained results are compared to the reference solution of 6-hydroxy-2,5,7,8-tetramethylchroman-2-carboxylic acid (TROLOX). The method entailing the reduction of DPPH^•^ was chosen due to the fact that the studied thiadiazole derivatives showed no absorption in the wavelength range within which the measurements are conducted. The obtained, averaged results yielded the curve of decreasing intensity of DPPH^•^ radical absorption at λ_max_ 519 nm relative to the increasing concentration of the respective analytes (Figure S8). The inclination angle of the decreasing absorption maximum curves indicates the speed of the reaction. In the respective points, the values of standard deviation (SD) were additionally established after three replicates of each experiment.

When comparing the obtained results, one should note the concentration ranges of the respective compounds necessary to reduce the fixed concentration of radicals. In all cases apart from TB, analytes concentrated at approx. 60–80 μM yielded a reduction of the fixed content of DPPH^•^ radicals by over 70% relative to the initial content thereof under the adopted experimental conditions, whereas in the case of TB the result was significantly smaller, approx. 7% (Figure S9). The poor activity of the latter, when compared to TS and TSF, results from its lack of substituent attached to the aromatic ring, which could take part in the reaction of reducing the radicals. After comparing the results obtained for the respective derivatives with TROLOX, we obtained the value of antioxidative potential expressed as a TROLOX equivalent (TE) (Table 4), which specifies how many times higher or lower the given compound’s potential is compared to the standard. In the case of the analyzed TS and TSF derivatives, the value oscillated at approximately one, which means that the two analogues are more active than any of the others including quercetin and the well-known flavonoid. The concentrations of the respective analytes needed to reduce the DPPH^•^ radicals by 50% of their initial concentration (IC_50_) (Table 4) to confirm the above observations.

The lower the concentration required to reduce a fixed number of radicals, the higher the antioxidative potential of the given compound. In the case of TSF, QUER, and TROL the values are similar and oscillate within the range of 41–45 μM (for 200 μM of the initial DPPH^•^ concentration). In the case of TS, the value is somewhat lower (34.74 μM), which confirms the higher antioxidative potential of this derivative (>1 TE). In the case of TB, the value is several times higher than the concentration of radicals in the analyzed sample, which disqualifies the compound as a potential antioxidant. In a previous paper all three analyzed analogues of 1,3,4 thiadiazoles were studied for their antioxidative properties using the TLC-DPPH method with the “dot-blot” test. As expected, the highest antioxidant activity was observed in TS, while TSF and TB demonstrated lower activity (TS > TSF > TB, respectively).

Natural compounds such as polyphenols or flavonoids owe their antioxidative properties to the presence of numerous functional, particularly hydroxyl groups in their structures [33], as the same can donate the electrons necessary for the reduction to occur. Based on structural analyses of the studied thiadiazole derivatives it could be preliminarily assumed that the TB derivative, unsubstituted on the aromatic ring, would show the lowest antioxidative activity, possibly none at all, which was later corroborated by the conducted experiments. The significantly higher TE and lower IC_50_ values (Table 4) observed for TS and TSF are most likely due to the presence in the aromatic rings of those derivatives of substituents capable of donating electrons.

### 2.4. Microbiological Analysis

In addition to the above-mentioned antioxidant properties of the selected studied analogues, it was decided to pay attention to the potential antifungal properties of some derivatives from this group of compounds. In the subsequent part of the study, biological tests on selected fungal species were conducted to determine the antimycotic properties of the analyzed analogues. *Candida* spp. are the microorganisms responsible for the most frequently encountered invasive fungal infections in highly developed countries. The widespread use of azoles and echinocandins has been accompanied by the emerging drug resistance of clinical isolates of *Candida* spp. [34]. Moreover, the conventional antifungal treatments for candidiasis based mainly on polyenes, azoles, and echinocandins are costly and can cause toxicity as well as side effects [35]. Thus, there is a need to develop novel antimycotic agents.

**Figure 9:**
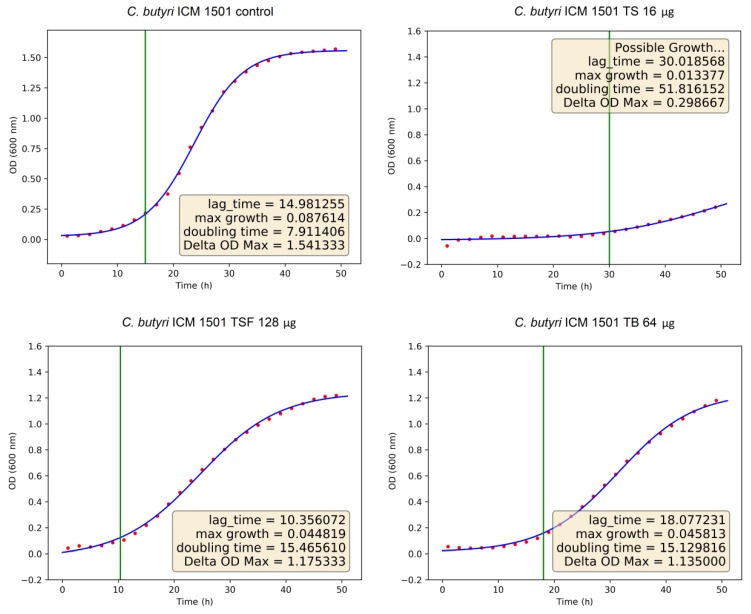
Sample regression curves of selected data showing the effect of examined 1,3,4-thiadiazoles on *Candida* species.

New pharmaceuticals such as 1,3,4-thiadiazoles can be a good alternative because of their antibacterial and antifungal properties [2]. The antimycotic potential of 2-amino-5-phenyl-1,3,4-thiadiazole (TB), 2-amino-5-(2-hydroxyphenyl)-1,3,4-thiadiazole (TS), and 2-amino-5-(2-hydroxy-5-sulfobenzoyl)-1,3,4-thiadiazole (TSF) was tested in this study against nine *Candida* species. The minimum inhibitory concentrations (MICs) of TB and TSF were similar for the tested organisms and ranged between 128 and >256 μg/mL with the exception of *Candida butyri* which showed growth inhibition at 64 μg/mL for TB (Figure 9).

The MICs of TS for five *Candida* species (*C. fructus*, *C. fragicola*, *C. butyri*, *C. shehatae*, and *C. fluviatilis*) ranged between 4 and 64 μg/mL while the other species tested between 128 and >256 μg/mL (Table S4). Based on the above results and the criteria proposed by Morales et al., TS exhibited strong antimycotic activity against *C. fructus*, *C. fragicola*, *C. butyri*, *C. shehatae*, and *C. fluviatilis* because its MIC values were lower than 100 μg/mL. An interesting observation is that *C. butyri* was the most sensitive to tested thiadiazole derivatives. Its maximum growth rate was reduced six-fold after the addition of TS to the growth medium at a concentration of 8 μg/mL (Figure 9). At the same time, the doubling time of the generation was six times longer compared to the control (Table S5). *Candida butyri* may be found in humans as opportunistic organisms awaiting the proper change in environmental conditions to become pathogens, such as in immunocompromised hosts. It was described as the etiological agent of the candidemia of an immunocompromised cancer patient [36]. Our observations were consistent with other studies whose results show that *Candida aaseri* strains (closely related to *C. butyri*) were susceptible to such antifungal agents as amphotericin B, flucytosine, ketoconazole, itraconazole, fluconazole, voriconazole, posaconazole, caspofungin, anidulafungin, and micafungin [36]. However, new resistant strains continue to be identified and it is therefore important to identify new compounds with antifungal potential.

## 3. Materials and Methods

All solvents were of 99% purity or higher (HPLC grade). Thiosemicarbazide, DPPH (2,2-diphenyl-1-picrylhydrazyl), TROLOX (6-hydroxy-2,5,7,8-tetramethylchroman-2-carboxylic acid), POCl_3_, benzoic, salicylic, and 5-sulfosalicylic acids were purchased from Sigma (Darmstadt, Germany). Quercetin, H_2_SO_4_, NH_3_, and NaOH were purchased from Avantor (Gliwice, Poland).

### 3.1. Isolation of Thiadiazole Derivatives

TB was synthesized *via* the method 1, namely the POCl_3_-catalyzed reaction between benzoic acid with thiosemicarbazide (Scheme 2) [37]. Benzoic acid was suspended in POCl_3_ and stirred at room temperature for 20 min after which an equimolar amount of thiosemicarbazide was added. The reaction mixture was heated up to 75 °C for 6 h and then cooled down to 35 °C. Excess POCl_3_ was quenched by adding water and the reaction mixture was refluxed for another 4 h at 105 °C. The cooled mixture was then brought to pH 8 using a saturated NaOH solution. The precipitate formed was filtered off, rinsed with water, dried and recrystallized from methanol. An identical procedure was applied for the synthesis of TS except that salicylic acid was used as the substrate.

TSF was synthesized according to method 2, namely the classical H_2_SO_4_ catalyzed route involving the use of thiosemicarbazide and appropriate 5-sulfosalicylic acid as the substrate (Scheme 2) [38,39].

An equimolar amount of 5-sulfosalicylic acid and thisoemicarbazide was dissolved in concentrated sulfuric acid and the mixture was heated up to 95 °C for 6 h. The mixture was then cooled down and basified to pH ~8 with use of the diluted ammonia. The excess solvent was removed under vacuum and the remaining mixture was left undisturbed. The precipitate formed was filtered off, rinsed with water, and recrystallized from ethanol.

All products were isolated with satisfactory yields and the purities were confirmed by LC-MS analyses. In particular, experiments comprising of the tandem mass spectrometry (MS/MS) analyses of the products obtained were performed. The values of the recorded daughter ions were consistent with those reported in the literature (Figure S10) [40].

### 3.2. pH Measurement

The TB, TS, and TSF (about 1 mg) samples were dissolved in 2 mL of distilled water, and then appropriate aliquots of this solution were added to 50 mL of distilled water. Due to a sparing solubility of the thiadiazoles tested, the concentrations of samples were not determined. In order to obtain pH 12, 0.1 M of NaOH was added to each sample. Next, 0.1 M of HCl acid was gradually added resulting in a desirable pH of aqueous TB, TS, and TSF solutions. The respective titration curve plots are given in Figure 2. All measurements were carried out at room temperature using the Elmetron CP-502 pH-meter.

### 3.3. Measurements of Electronic Absorption and Fluorescence Spectra

The electronic absorption spectra of the selected compounds in selected organic solvents were recorded using a double-beam UV–Vis spectrophotometer Cary 300 Bio Varian (Middelburg, The Netherlands) equipped with a thermostatted cuvette holder with a 6 × 6 multicell Peltier block. The temperature was controlled with a thermocouple probe (Cary Series II from Varian) placed directly in the sample.

The UV–Vis spectra were recorded on Cary 300 Bio (Varian) instrument. All measurements were carried out in a thermostatted cuvette holder with a 6 × 6 multicell Peltier block. The thermocouple probe (Cary Series II from Varian) was applied for the temperature control.

Cary Eclipse spectrofluorometer (Varian) was applied for the recording of fluorescence excitation, emission, and synchronous spectra. All measurements were carried out at 22 °C. All the fluorescence spectra were recorded with 0.5 nm resolution together with the lamp and photomultiplier spectral characteristics corrections. Resonance light scattering (RLS) measurements were carried out according to the previously reported protocol [25,37] with synchronous scanning of both the excitation and emission monochromators (there was no interval between excitation and emission wavelengths) and a spectral resolution of 1.5 nm. Grams/AI 8.0 software (Thermo Electron Corporation; Waltham, Massachusetts, United States) was applied for analysis of the data recorded.

### 3.4. Methodology and Calculation of Dipole Moments

Analytical grade methanol, DMSO, dimethylformamide (DMF, ethanol, propan-2-ol, butan-1-ol, acetonitrile, toluene, chloroform, and cyclohexane were used as solvents. The TB, TS, and TSF (about 1 mg) were made up in 1 mL of each solvent. The absorbance intensity was adjusted by the addition of an appropriate aliquot of TB, TS, and TSF into 2 mL of solvent. The concentration in several samples remained undetermined due to a poor solubility of the thiadiazoles (TB, TS, and TSF were readily soluble only in DMSO and DMF). The molar concentrations of TB, TS, and TSF dissolved in DMSO and DMF were as follows: C1 = 0.056 mM, C2 = 0.055 mM, C3 = 0.038 mM, C4 = 0.062 mM, C5 = 0.056 mM, C6 = 0.036 mM.

In order to estimate the dipole moments of TB, TS, and TSF molecules, two methods were used depending on the internal electric field effect.

#### 3.4.1. Method 1

In method 1, the effect of polarization dependence and hydrogen bonding effects with solvents can be expressed by the function $E_{T}^{N}$ which was first proposed by Reichardt [28,38,39]. A normalized E_T_(30) value, specifically the microscopic solvent polarity ($E_{T}^{N}$) is useful in measuring solvent polarity by solvatochromic methods using betaine dye as a probe solute. $E_{N}^{T}$ is defined by Equation (1) using water ($E_{T}^{N}$ = 1) and tetramethylsilane ($E_{T}^{N}$ = 0) as the reference solvents:

(1) $$E_{T}^{N}=\frac{E_{T}\left(\mathrm{solvent}\right)-E_{T}\left(\mathrm{TMS}\right)}{E_{T}\left(\mathrm{water}\right)-E_{T}\left(\mathrm{TMS}\right)}=\frac{E_{T}\left(\mathrm{solvent}\right)-30.7}{32.4}$$

The change in dipole moment can be determined by Equation (2):

(2) $$\Delta \mu =\mu_{e}-\mu_{g}=\sqrt{\frac{81m}{{\left(6.2/a_{0}\right)}^{3}11307.6}}$$

where *m* is the slope obtained from a linear plot of Stokes shift against the microscopic solvent polarity $E_{T}^{N}$. The values of E_T_(30) and $E_{T}^{N}$ for the solvent are presented in Table S2.

#### 3.4.2. Method 2

In method 2 the ground and excited state dipole moments were calculated based on Kawski and Bilot equations [28,29]. By employing Onsager’s reaction field theory and simple quantum mechanical second order perturbation theory of absorption ($v_{a}$) and fluorescence ($v_{f}$) band shift in different solvents, they receive the two following relations:

(3) $$v_{a}-v_{f}=m_{1}F\left(\varepsilon ,n\right)+const$$

(4) $$v_{a}+v_{f}=-m_{2}F\left(\varepsilon ,n\right)+2g\left(n\right)+const$$

The expressions giving *F*(*ε*,*n*) and *F*(*ε*,*n*) + 2*g*(n) are provided by Equations (5) and (6). In these equations, n is the refractive index and *ε* is the dielectric constants of the solvent, respectively.

(5) $$F\left(\varepsilon ,n\right)=\frac{2n^{2}+1}{n^{2}+2}\left(\frac{\varepsilon -1}{\varepsilon +2}-\frac{n^{2}-1}{n^{2}+2}\right)$$

(6) $$g\left(n\right)=\frac{3}{2}\frac{n^{4}-1}{{\left(n^{2}+2\right)}^{2}}$$

Assuming that the symmetry of the investigated solute molecule remains unchanged upon electronic transition, and the ground and excited-state dipole moment are parallel we obtain the following equations:

(7) $$\mu_{g}=\frac{m_{2}-m_{1}}{2}{\left(\frac{hca_{0}^{3}}{2m_{1}}\right)}^{1/2}$$

(8) $$\mu_{e}=\frac{m_{1}+m_{2}}{2}{\left(\frac{hca_{0}^{3}}{2m_{1}}\right)}^{1/2}$$

(9) $$\mu_{e}=\frac{m_{1}+m_{2}}{m_{2}-m_{1}}\mu_{g};(m_{2}>m_{1})$$

where $\mu_{g}$ and $\mu_{e}$ are the ground and excited-state dipole moments of the solute molecule, h is Planck’s constant, c is the velocity of light in vacuum, and $a_{0}$ is the Onsager cavity radius. The value of the solute cavity radius was calculated based on the Van der Waals volumes (Equation (10)) according to Edward’s method [40].

(10) $$V=\frac{4}{3}\pi a_{0}^{3}$$

The calculation of molar volume V was performed applying ACD/ChemSkech software. Slopes m_1_ and m_2_ were calculated by plotting Stokes shifts and v_a_ + v_f_ against the bulk solvent polarity functions *F*(*ε*,*n*) and *F*(*ε*,*n*) + 2*g*(n), respectively; where, $v_{a}$ is the absorption maximum in cm^−1^ and $v_{f}$ is the fluorescence maximum in cm^−1^.

The change in the dipole moment can be determined as (Equation (11)):

(11) $$\Delta \mu =\mu_{e}-\mu_{g}$$

The value of the refractive index n, dielectric constant ε of the solvents, functions *F*(*ε*,*n*), and *F*(*ε*,*n*) + 2*g*(n) of the solvents are presented in Table S2.

### 3.5. Fluorescence Lifetime Measurements

Multifrequency cross-correlation phase and modulation K2 fluorometer (ISS, Champaign, IL, USA) was applied for the fluorescence lifetime measurements. A continuous wave 280 nm light emitting diode (LED; model 73115) was applied for the excitation and the decays were recorded in a 10 × 10 mm quartz cuvette. The fluorescence signal was recorded using a monochromator set at the maximum emission (±10 nm). These emissions were of 405 nm for TSF, 382 nm for TB, and 415 nm for TS. Measurements were performed for 15 modulation frequencies ranging from 2 to 200 MHz. The phase shift and demodulation ratios were referenced to the aqueous solution of Ludox@ from Aldrich (Darmstad, Germany) without the emitter filter. ISS Vinci 2.0 software was used for the data analysis utilizing a multiexponential decay model with discrete fluorescence lifetime components (Equation (12))

(12) I(λ,t)= ∑iαie−tτi= ∑ifi(λ)τie−tτi

where *I*(*λ*,*t*) is the fluorescence intensity, *α_i_* is the preexponential factor, and *f_i_* is the fractional contribution of each fluorescence lifetime component. Minimization of the reduced χ^2^ value together with the residual distribution of the experimental data allowed for achieving the best-fit parameters.

The presented results were calculated as an average of three to five repetitions of fluorescence lifetime measurements.

### 3.6. Computational Details

The DFT calculations were made using the Gaussian 09 package [41], B3LYP exchange-correlation functional [42], aug-cc-pVDZ basis set [43], and very dense grids. The solvent effects were modeled according to the polarizable continuum model (PCM) [44]. Sodium cation and chlorine anion were used as counterions in the case of charged species. The excited state treatment was carried out using a standard random-phase approximation (RPA) approach to the TD-DFT formalism [45]. The influence of the solvent on energetics was determined both according to the standard linear response (LR) approximation and more exact state-specific (SS) approach [46]. In the case of TS dimer, only the LR results are provided as the SS procedure was not converging.

### 3.7. Analysis of Antioxidative Properties

Increasing concentrations of ethanolic solutions of tested thiadiazol derivatives (TB, TS, TSF) were applied to the following wells of the 96-well plate, so that their final concentration in 200 μL of the total volume of each sample was within the range of 0–60 μM. Samples of reference compounds, namely quercetin (QUER) and TROLOX (TROL), were prepared in the same way and their final concentrations in 200 μL of the total volume of each sample was within the range of 0–75 μM. Immediately before the measurement, all wells were supplemented with 1 mM of an ethanolic solution of DPPH^•^ radicals, so that their final concentration in each well was 200 μM. The plate was shaken on the internal shaker of the Tecan Infinite 200 microplate reader (Tecan Austria GmbH, Grödig/Salzburg, Austria) for 10 s to ensure thorough mixing of reagents in all wells, and spectrophotometric measurements started in the range of 500–550 nm with a 1 nm wavelength step at 25 °C for 30 min. The results obtained were the average of five exposures of each sample to a beam of light. All experiments were repeated three times.

### 3.8. Microbiological Analysis

#### 3.8.1. Antifungal Agents

The antifungal agents (TB, TS, TSF) were dissolved in 100% DMSO, at a final concentration of 1000 μg/mL. Ten 2-fold serial dilutions were prepared in Roswell Park Memorial Institute (RPMI)-1640 medium (supports cell growth in research and biomanufacturing was developed by George E. Moore, Robert E. Gerner, and H. Addison Franklin in 1966 at Roswell Park Memorial Institute, from where it derives its name.), with the final drug concentrations ranging from 256 to 0.125 μg/mL of examined agents, in accordance with CLSI guidelines [47].

#### 3.8.2. Fungal Strains and Culture Method

*Candida crusei* (Polish isolate), *C. fructus* (JCM 1513), *C. fragicola* (JCM 1589), *C. butyri* (JCM 1501), *C. tropicalis* (ATCC 1369), *C. shehatae* (ATCC 22984), *C. fluviatilis* (CBS 6776), *C. freyschussi* (CBS 3562), and *C. parapsilopsis* (DSM 70125) strains were used in this study. The strains were inoculated onto Sabouraud dextrose agar (BTL, Poland, Łódź) plates from glycerol stocks and incubated at 30 °C for 24 h. They were then subcultured on the same medium for a further 24 h at 30 °C. The yeast inocula were prepared by diluting the overnight culture with 0.9% NaCl to 1–5 × 10^6^ a colony-forming unit CFU/mL compared to the isolate density standard corresponding to 0.5 in the McFarland scale.

#### 3.8.3. Susceptibility Testing

Thiadiazole derivative MICs were determined using the broth microdilution method described in CLSI M27-A2 for yeasts. The MIC was defined as the lowest concentration of antifungal agent that produced a prominent decrease in optical turbidity when compared with the control. The antimicrobial activity of thiadiazole derivatives was interpreted as strong/good activity (MIC: <100 μg/mL), moderate activity (MIC: 100–500 μg/mL), weak activity (MIC: 500–1000 μg/mL), and inactive/no antimicrobial effect (MIC: >1000 μg/mL) according to the criteria proposed by Morales et al. [48].

Fungal inocula (30 μL) were added to each well on a sterile, 100-well flat-bottomed microtiter plate containing the test concentration of thiadiazole derivatives (350 μL/well). Each concentration was tested in triplicate for each organism. Two wells containing a fungal suspension with no antifungal agent (compound-free growth control) and two wells containing only growth media (background control) were included in this plate. ODs (optical density) were measured for 24 h at 30 °C using a multi-detection microplate reader (Bioscreen C system, Labsystem, Helsinki, Finland) at 600 nm and were automatically recorded every 2 h for each well. Turbidimetric growth curves were obtained depending on the changes in the OD of fungal growth for each antifungal agent concentration and the compound-free growth control. Growth curve parameters (i.e., max specific growth rate, lag time, doubling time) were determined using the PYTHON script according to Hoeflinger et al. [49].

## 4. Conclusions

The fluorescence spectroscopy experiments presented in this paper indicate the emergence in the fluorescence emission spectra of the selected 1,3,4-thiadiazole analogues (TS) of the effect of dual fluorescence in varying pH media or for varying concentrations of the analyzed compounds. The effect studied with the use of fluorescence spectroscopy (with the RLS technique and excitation spectra) and by measuring the respective fluorescence lifetimes revealed a strong dependence on the phenomenon of molecular aggregation of the analyzed analogues. A very important role in the context of the observed fluorescence effects was also played by the structure of the substituent system (in the resorcylic group) which determined the type of aggregative interactions. The studies of TS fluorescence spectra indicated the emergence of the dual fluorescence effect for this compound within the pH range of 1 to ~4/5. Based on the exciton splitting theory, the effects can be associated with the prevalence of card pack aggregation. Moreover, the calculations pertaining to the changes in dipole moment between the ground and excited states of the analyzed molecules, performed with two different methods, as well as quantum-mechanical TD-DFT calculations very clearly suggest that the possibility of intermolecular CT may in fact be the main cause of the effects related to dual fluorescence in the case of TS as well as highly concentrated TSF. The effects of fluorescence emission quenching or the noticeable (albeit slight) reduction of the mean fluorescence lifetime, confirm the participation of aggregation effects as a factor necessary for CT to occur. Charge transfers seemed to be particularly evident in the TS molecule, it was also in this compound that the UV–Vis, fluorescence excitation spectra within the pH range of 1–4/5, revealed the strongest aggregation effects. Moreover, CT effects must be associated with the correct conformation of the molecule (i.e., the –OH group in the resorcylic ring), positioned on the side of the nitrogen atom in the thiadiazole ring, to allow the formation of correct hydrogen bonds.

In case of the investigated 1,3,4-thiadiazole analogues a second mechanism for their dual fluorescence cannot be excluded. In particular an excitation of the enolic tautomer may lead to the formation of its excited keto tautomer, and the subsequent fluorescence emission from such excited keto form of the compound. In order to verify this hypothesis more in-depth studies will be carried out in the future.

The preliminary microbiological studies also clearly revealed the influence of the substituent system on the antimycotic properties of the analyzed analogues. The most promising results were obtained for TS and TSF in which the effects of dual fluorescence were also observed. It is noteworthy that the studies discussed above may facilitate a quick analysis of the structure of the studied molecule in any biological or crystalline system, which may allow the whole group of analogues to be used as excellent fluorescence probes, highly sensitive to changing conditions in the given environment.

Furthermore, one will also be able to exactly determine which molecular form and which particular conformation is responsible for a given aspect of the biological activity displayed by this group of analogues. At this point it is also worth emphasizing that due to their interesting photophysical properties the thiadiazoles selected in this research represent a group of remarkably useful molecular probes, whose properties easily change depending on environmental conditions.

## Figures and Tables

**Table 1:** Slope m from Stokes shift versus microscopic polarity function, change in dipole moment ∆μ, and coefficient of determination r^2^ for **TB**, **TS**, and **TSF**.

Compound	m	Δμ (D)	r^2^
TB	3554.36	2.37	0.82
TS	8976.70	3.75	0.91
TSF	8729.69	4.00	0.89

**Table 2:** m_1_ and m_2_ determined by Stokes shift against function *F*(*ε*,*n*), and m_2_ determined by ($v_{a}$ + $v_{f}$) against *F*(*ε*,*n*) + 2*g*(n), ground state $\mu_{g}$, excited state $\mu_{e}$, change in dipole moment ∆μ, and correlations factor r^2^ determined for both figures for compounds TB, TS, and TSF.

Compound	$V_{m}\left[\frac{cm^{3}}{mol}\right]$	a_0_ [Å]	m_1_	m_2_	$\frac{\mu_{e}}{\mu_{g}}$	$\mu_{e}\;(D)$ ^a^	$\mu_{g}\;(D)$ ^b^	Δμ (D) ^c^	r^2^
TB	132.9 ± 3.0	3.75	1852.26	−9217.88	1.50	9.30	6.19	3.11	0.76:0.75
TS	131.3 ± 3.0	3.73	3748.56	−18,746.02	1.50	13.21	8.81	4.40	0.91:0.95
TSF	154.1± 3.0	3.94	3948.56	−16,703.42	1.62	12.80	7.91	4.90	0.82:0.95

1 D = 10^−18^ statC · cm; ^a^ Value calculated from the plot of Stokes shift vs. *F*(*ε*,*n*), ^b^ Value calculated from the plot of $v_{a}$ + $v_{f}$ shift vs. *F*(*ε*,*n*) + 2*g*(n). ^c^ The dipole change calculated from the experimental values of μ_g_ and μ_e_.

**Table 3:** Predictions for low-lying excited states of various forms of the TS molecule in water. Energetics given in nm scale, dimensionless oscillatory strengths for the absorption process given in parentheses, SS approach-based energies given in italics. Intense absorbing states and the lowest (emitting) state reported.

Form	Vertical Absorption	Adiabatic Absorption/Emission	Vertical Emission
TS^−^	387/*367* (0.38)	417/*389*	451/*421*
	283/*279* (0.22)		
TSH	334/*315* (0.64)	363/*337*	397/*368*
	290/*285* (0.17)		
TSH_2_^+^	326/*307* (0.325)	355/*330*	377/*348*
	283/*279* (0.29)		
(TSH)_2_	331 (0.34)	382	449
	327 (0.22)		
	323 (0.31)		
	286 (0.28)		

**Table 4:** Potential of the tested derivatives and selected reference compounds 6-hydroxy-2,5,7,8-tetramethylchroman-2-carboxylic acid expressed as TROLOX equivalents (TE) (μM antioxidant/μM TROLOX) and as IC_50_ values. Results obtained after 30 min of DPPH^•^ (200 μM) radicals reduction reaction at 25 °C. QUER: quercetin.

	TE ± (SD)	IC_50_ DPPH^•^ (μM) ± (SD)
TB	0.03 ± 0.00	1094.64 ± 14.8
TS	1.10 ± 0.10	34.74 ± 0.29
TSF	0.90 ± 0.07	43.62 ± 0.40
QUER	0.84 ± 0.06	45.83 ± 0.43
TROL	1.00 ± 0.09	41.76 ± 0.38

## Supplementary Materials

Supplementary materials can be found at https://www.mdpi.com/1422-0067/20/21/5494/s1. Figure S1: Fluorescence excitation spectra of TB (panel A) and TSF (panel B) dissolved in H2O at different pH. The spectra were measured at room temperature; Figure S2: Fluorescence excitation spectra of TS dissolved in H2O at different pH (panel A pH = 2, panel B pH = 3, panel C pH = 4, panel D pH = 5, panel E pH = 6, panel F pH = 7). The spectra were measured at room temperature; Figure S3: Intensity of resonance light scattering spectra in 436 nm of TB (black circles), TS (red circles) and TSF (blue circles) relative tochange in pH; Figure S4: The ratio of the maximum fluorescence intensity at 434/492 nm for TS and TSF dissolved in butan-1-ol in different excitation depending on the changes in concentration; Figure S5: Stokes shift variation with normalized value of solvent polarity E_T^N for TB, TS and TSF for various solvent (1—cyclohexane, 2—toluene, 3—chloroform, 4—ethyl acetate, 5—butan-1-ol, 6—propan-2-ol, 7—ethanol, 8—DMF, 9—DMSO, 10—methanol, 11—acetonitrile); Figure S6: Stokes shift versus F(ε,n) for (panel A), v_a + v_f versus F(ε,n) + 2g(n) (panel B), for TB, TS, TSF dissolved in different solvents (1—cyclohexane, 2—toluene, 3—chloroform, 4—ethyl acetate, 5—butan-1-ol, 6—propan-2-ol, 7—ethanol, 8—DMF, 9—DMSO, 10—methanol, 11—acetonitrile); Figure S7: Fluorescence lifetimes ( ) and fractional intensities (%) measured for TSF, TS and TBrelative topH. Panel A—the main fluorescence lifetime component and panel B—the main fluorescence lifetime component + the second component when present; Figure S8: DPPH• radicals (200 μM) absorption intensity decrease at λmax 519 nm in the presence of increasing concentration of tested compounds after 30 min of reaction at 25 °C; Figure S9: Percentage of reduced DPPH• radicals under the influence of increasing concentration of compounds tested after 30 minutes of reaction at 25 °C. The measurements were taken at λ max 519 nm; Figure S10: Tandem mass spectrometry (MS/MS) of the thiadiazole derivatives studied: (A) TB, (B) TS, and (C) TSF. The corresponding MS-chromatographic traces are given in inserts. The MS/MS measurements were carried out using the collision energy of −30 eV; Table S1: Spectroscopic data. Maximum absorbance, maximum fluorescence and Stokes shift in cm-1 for TB, TS, TSF; Table S2: Physical constants of solvents. The average dipole molecular polarizability α, dielectric constant ε, index of refraction n, functions, F(ε,n) and F(ε + n) + 2g(n) of the solvents; Table S3: Lifetimes (τ) and fractional intensities (f) measured for TSF, TS and TB depending for pH; Table S4: Thiadiazole derivatives MICs for 9 Candida species; Table S5: MIC curves interpolations of thiadiazole derivatives against Candida species.
