# Supplementary material for: Non-Typical Fluorescence Effects and Biological Activity in Selected 1,3,4-thiadiazole Derivatives: Spectroscopic and Theoretical Studies on Substituent, Molecular Aggregation, and pH Effects
Source: Int J Mol Sci. 2019 Nov 4;20(21):5494. doi: 10.3390/ijms20215494 (PMC6862516; doi:10.3390/ijms20215494)
Supplement: Supplementary file 1 [file ijms-20-05494-s001.pdf]

Non-Typical Fluorescence Effects and Biological Activity in Selected 1,3,4-thiadiazole Derivatives – Spectroscopic and Theoretical Studies on Substituent, Molecular Aggregation and pH Effects

Iwona Budziak [3], Dariusz Karcz (ORCID: https://orcid.org/0000-0002-9848-2508) [2], Marcin Makowski [4], Kamila Rachwał [5], Karolina Starzak (ORCID: https://orcid.org/0000-0002-0343-5363) [2], Alicja Matwijczuk (ORCID: https://orcid.org/0000-0002-9626-4013) [1], Beata Myśliwa-Kurdziel [8], Anna Oniszczuk [6], Maciej Combrzyński[9], Anna Podleśna (ORCID: https://orcid.org/0000-0001-5652-9991) [7] and Arkadiusz Matwijczuk (ORCID: https://orcid.org/0000-0003-2630-120X) [1,*]

[1] Department of Biophysics, University of Life Sciences in Lublin, Akademicka 13, 20-950 Lublin, Poland; arkadiusz.matwijczuk@up.lublin.pl, alicja.matwijczuk@up.lublin.pl

[2] Department of Analytical Chemistry (C1), Faculty of Chemical Engineering and Technology, Cracow University of Technology, Warszawska 24, 31-155 Cracow, Poland; dkarcz@chemia.pk.edu.pl, kstarzak@chemia.pk.edu.pl

[3] Department of Chemistry, University of Life Sciences in Lublin, 20-950 Lublin, Poland; iwona.budziak@up.lublin.pl

[4] Department of Theoretical Chemistry, Faculty of Chemistry, Jagiellonian University, Gronostajowa 2, 30-387 Kraków, Poland; makowskm@chemia.uj.edu.pl

[5] Department of Biotechnology, Microbiology and Human Nutrition, University of Life Sciences in Lublin, Skromna 8, 20-704 Lublin, Poland; kamila.rachwal@up.lublin.pl

[6] Department of Department of Inorganic Chemistry, Medical University in Lublin, Lublin, Poland; anna.oniszczuk@umlub.pl

[7] Department of Plant Nutrition and Fertilization, Institute of Soil Science and Plant Cultivation - State Research Institute, Puławy, Poland; ap@iung.pulawy.pl

[8] Department of Plant Physiology and Biochemistry, Faculty of Biochemistry, Biophysics and Biotechnology, JagiellonianUniversity,Cracow, Poland; b.mysliwa-kurdziel@uj.edu.pl

[9] Department of Thermal Technology and Food Process Engineering, University of Life Sciences in Lublin, Lublin, Poland; maciej.combrzynski@up.lublin.pl

* Correspondence: arkadiusz.matwijczuk@up.lublin.pl; Tel: +(48 81) 445 69 37, Fax: +(48 81) 4456684; Department of Physics, University of Life Sciences in Lublin 20-950 Lublin, Poland

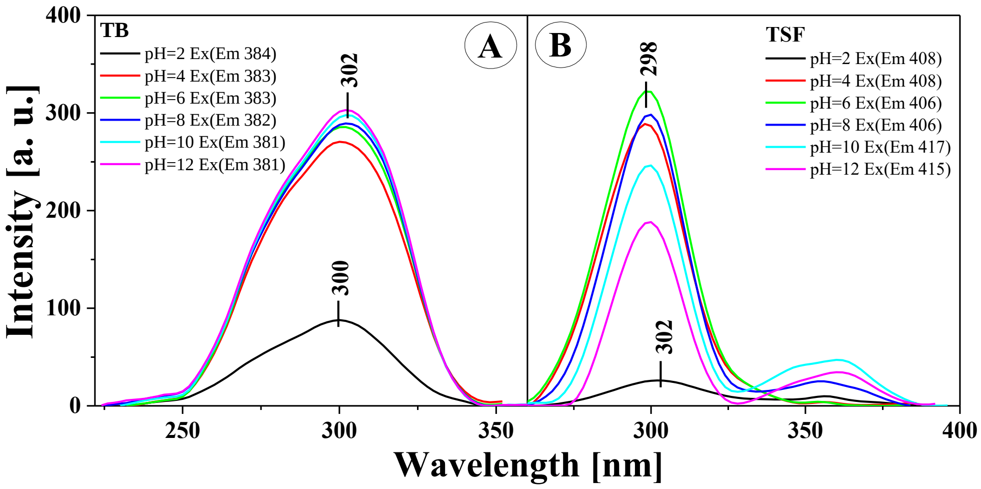

Figure S1. Fluorescence excitation spectra of **TB** (panel A) and **TSF** (panel B) dissolved in H_2O at different pH. The spectra were measured at room temperature.

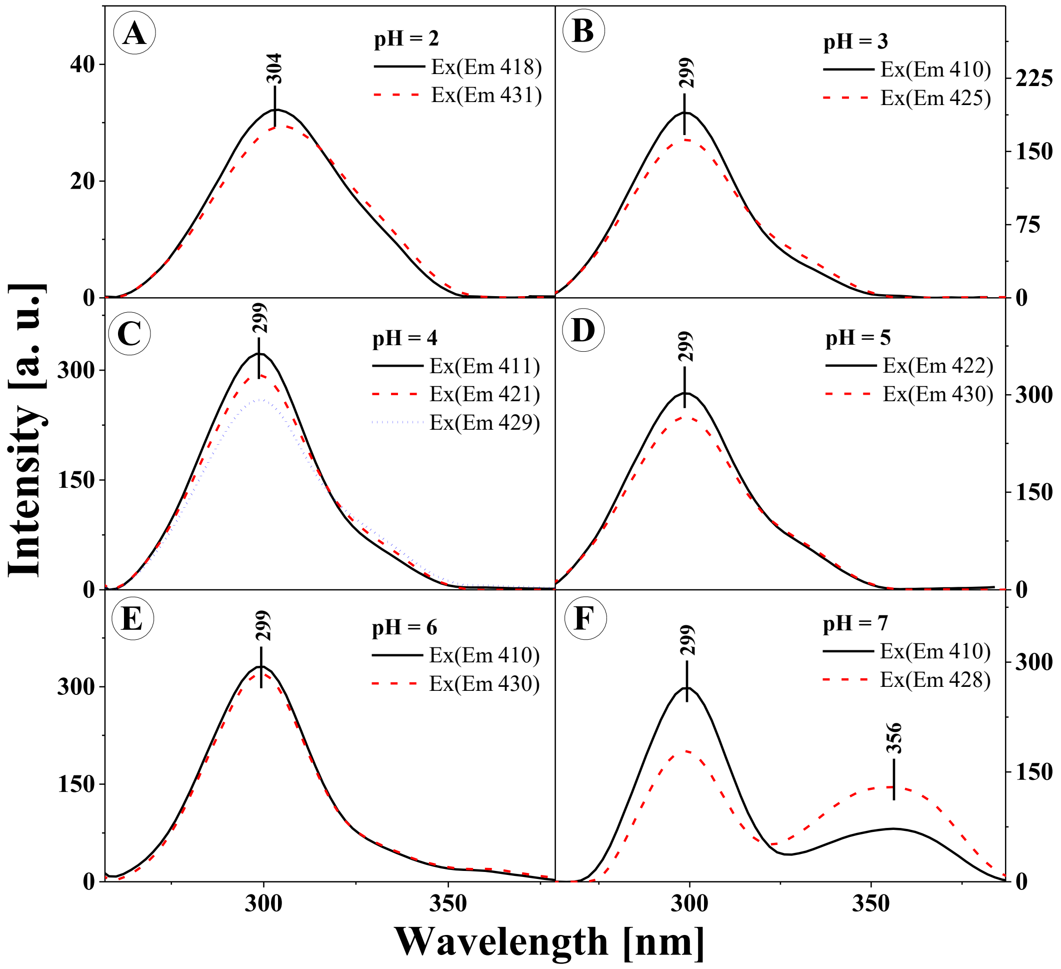

Figure S2. Fluorescence excitation spectra of TS dissolved in H_2O at different pH (panel A pH=2, panel B pH=3, panel C pH=4, panel D pH=5, panel E pH=6, panel F pH=7). The spectra were measured at room temperature.

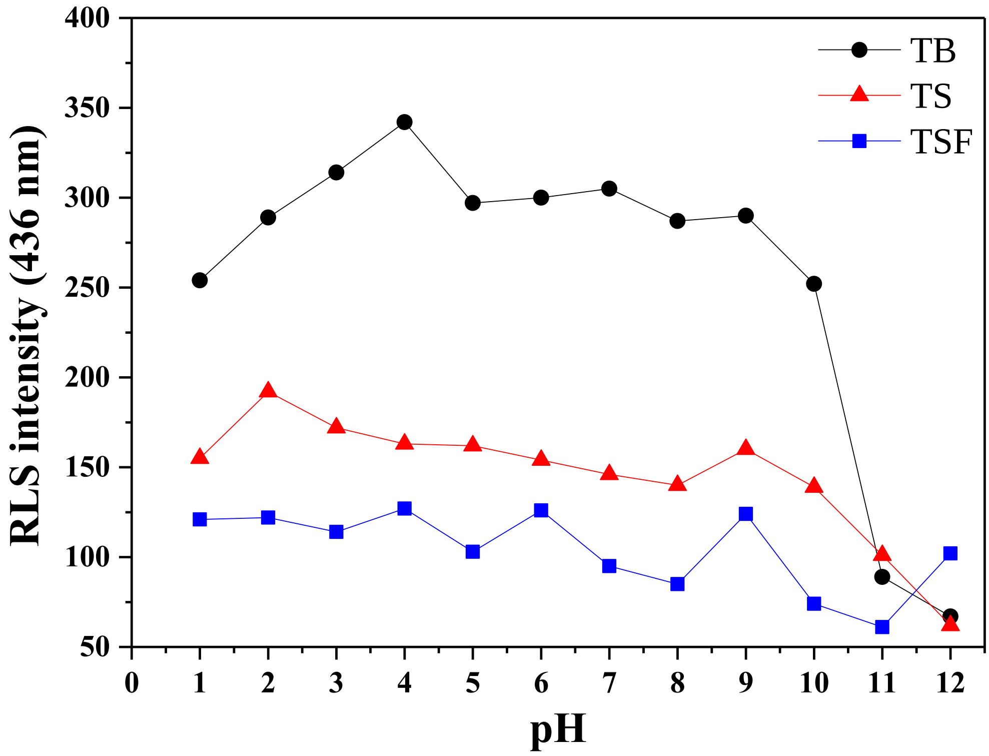

Figure S3. Intensity of resonance light scattering spectra in 436 nm of TB (black circles), TS (red circles) and TSF (blue circles) relative tochange in pH.

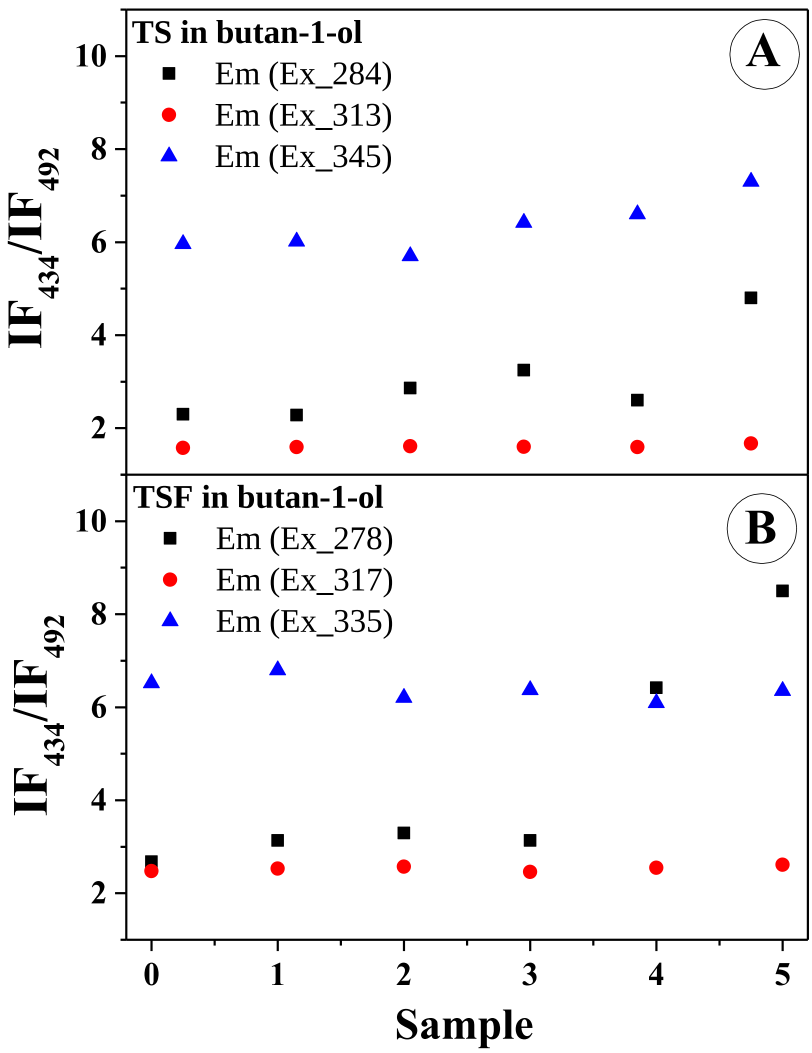

Figure S4. The ratio of the maximum fluorescence intensity at 434/492 nm for **TS** and **TSF** dissolved in butan-1-ol in different excitation depending on the changes in concentration.

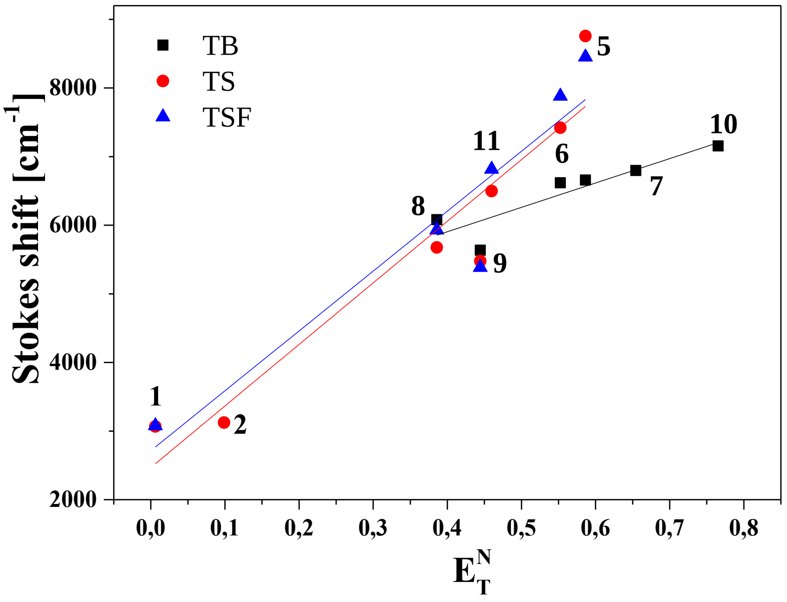

Figure S5. Stokes shift variation with normalized value of solvent polarity E_T^N for **TB**, **TS** and **TSF** for various solvent (1 - cyclohexane, 2 - toluene, 3 - chloroform, 4 - ethyl acetate, 5 - butan-1-ol, 6 - propan-2-ol, 7 - ethanol, 8 - DMF, 9 - DMSO, 10 - methanol, 11 - acetonitrile).

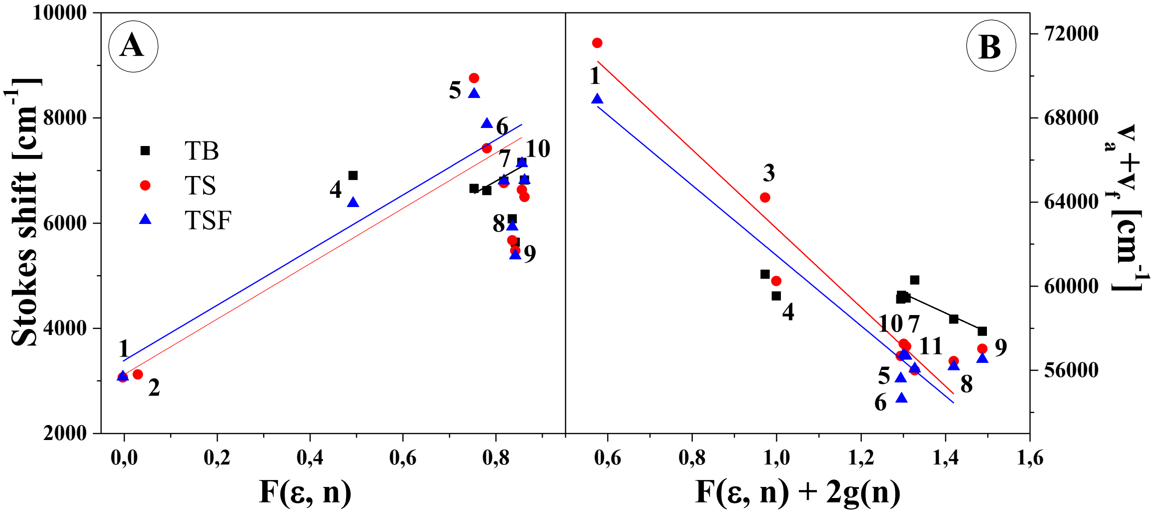

Figure S6. Stokes shift versus $F(\varepsilon,n)$ for (panel A), $v_a + v_f$ versus $F(\varepsilon,n)$+2$g(n)$ (panel B), for **TB**, **TS**, **TSF** dissolved in different solvents (1 - cyclohexane, 2 - toluene, 3 - chloroform, 4 - ethyl acetate, 5 - butan-1-ol, 6 - propan-2-ol, 7 - ethanol, 8 - DMF, 9 - DMSO, 10 - methanol, 11 - acetonitrile).

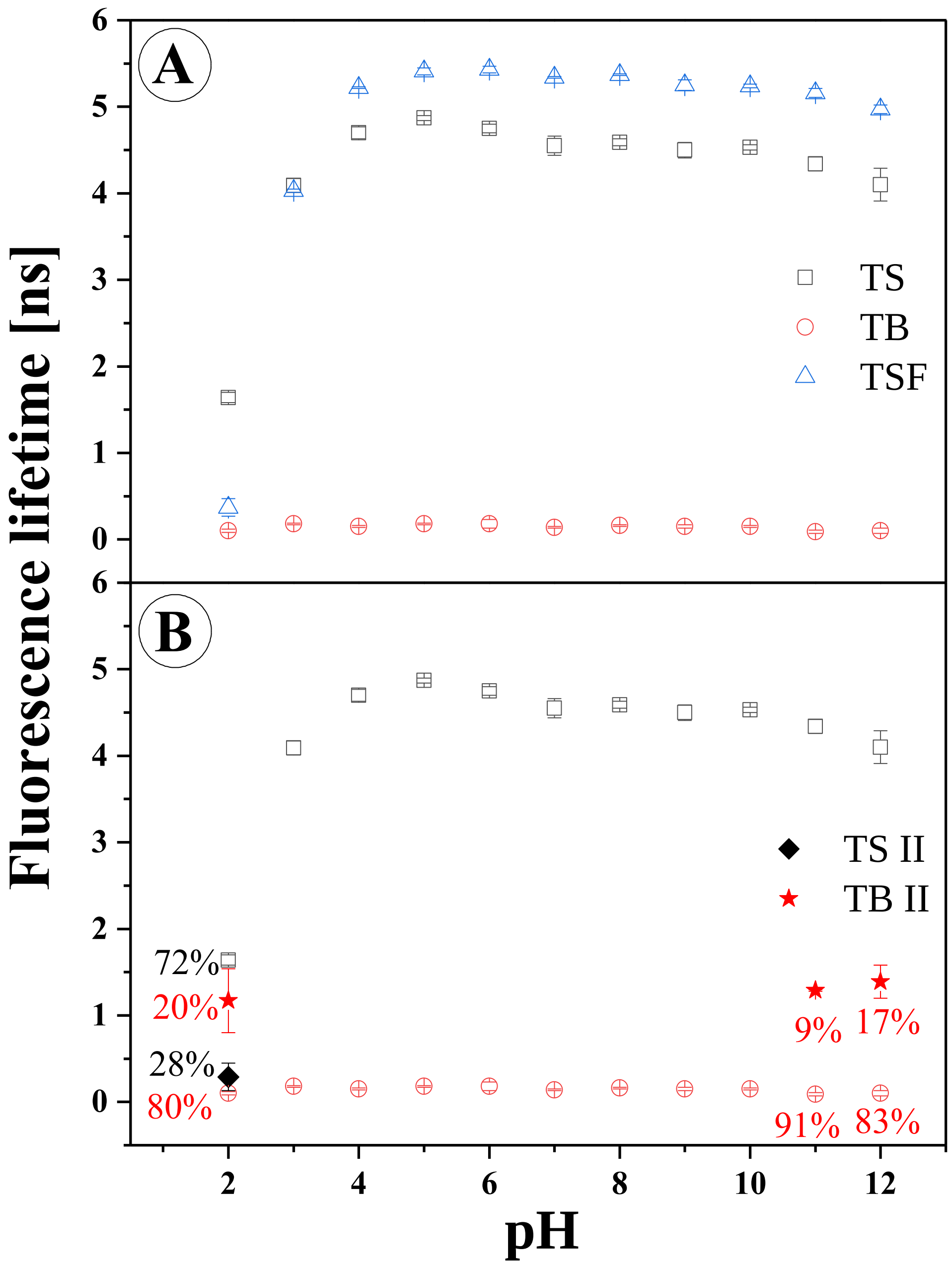

Figure S7. Fluorescence lifetimes (τ) and fractional intensities (%) measured for **TSF**, **TS** and **TB**relative topH. Panel A – the main fluorescence lifetime component and panel B - the main fluorescence lifetime component + the second component when present.

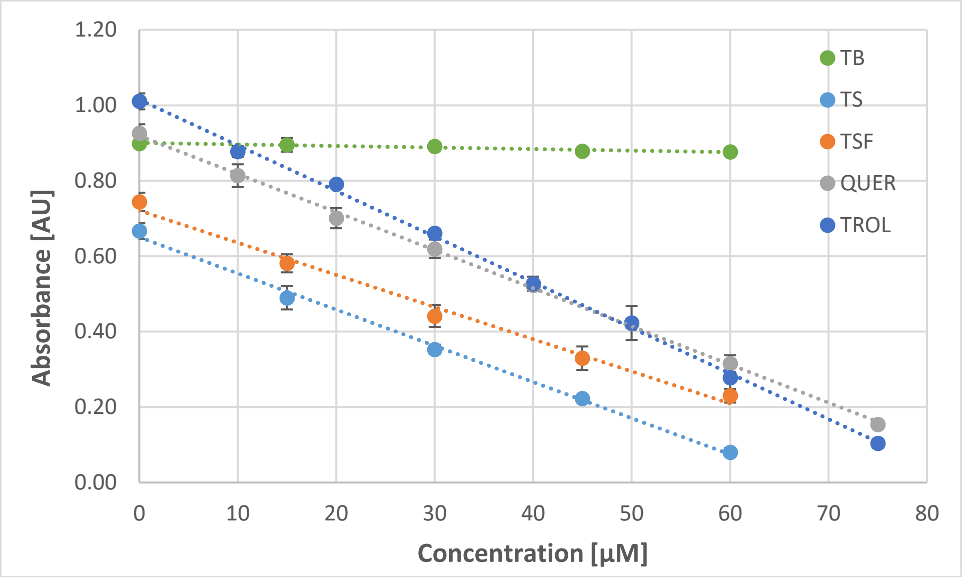

Figure S8. DPPH• radicals (200 μM) absorption intensity decrease at λ_{max} 519 nm in the presence of increasing concentration of tested compounds after 30 min of reaction at25 °C.

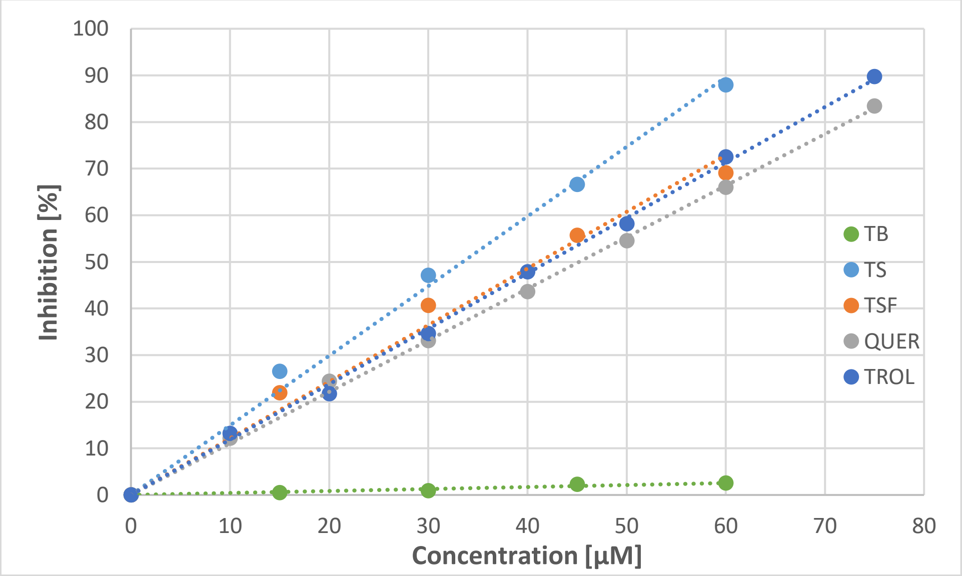

Figure S9. Percentage of reduced DPPH• radicals under the influence of increasing concentration of compounds tested after 30 minutes of reaction at 25 °C. The measurements were taken at λ_{max} 519 nm.

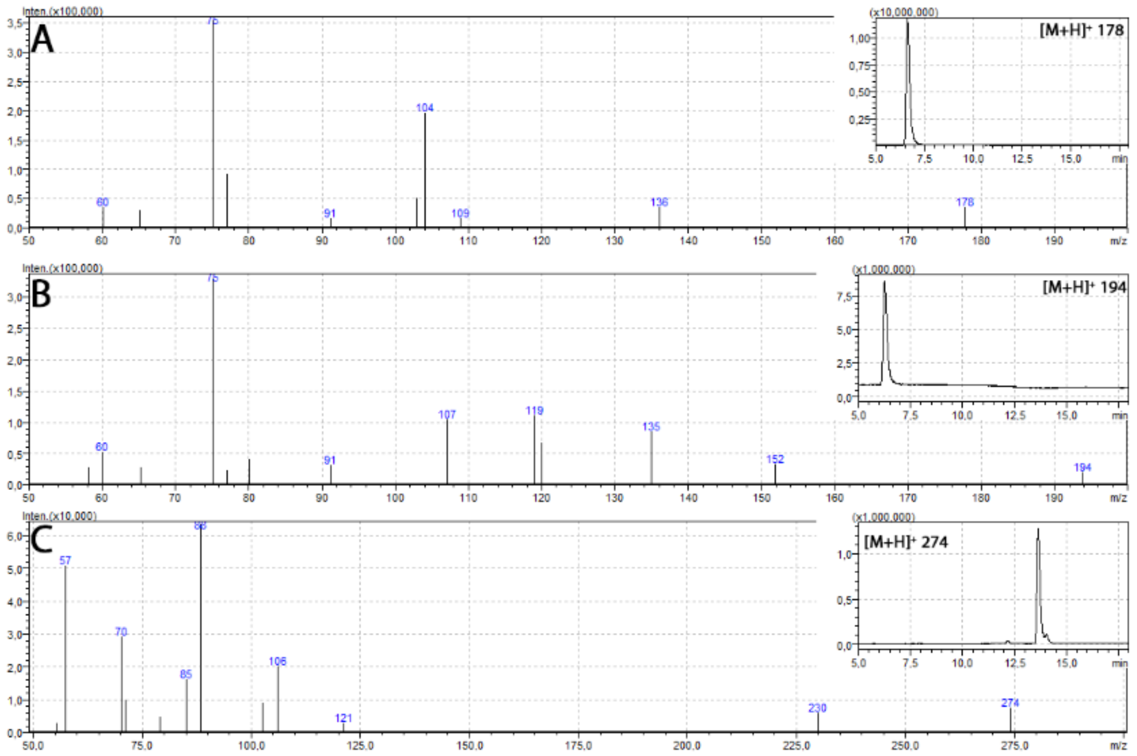

Figure S10. Tandem mass spectrometry (MS/MS) of the thiadiazole derivatives studied: A) **TB**, B) **TS**, and C) **TSF.** The corresponding MS-chromatographic traces are given in inserts. The MS/MS measurements were carried out using the collision energy of -30 eV.

Table S1. Spectroscopic data. Maximum absorbance, maximum fluorescence and Stokes shift in cm^{-1} for TB, TS, TSF.

Solvents	Absorbance [cm^{-1}]			Fluorescence [cm^{-1}]			Stokes shift [cm^{-1}]		
	TB	TS	TSF	TB	TS	TSF	TB	TS	TSF
**Methanol**	33333	31949	31949	26 178	25 316	24 814	7 155	6 633	7 135
**Acetonitrile**	33557	31250	31447	26 738	24 752	24 631	6 819	6 498	6 816
**Ethanol**	33113	31949	31746	26 316	25 189	24 938	6 797	6 760	6 808
**DMSO**	31746	31250	30960	26 110	25 773	25 575	5 636	5 477	5 385
**Propan-2-ol**	33003	32051	31746	26 385	24 631	23 866	6 618	7 420	7 880
**DMF**	32258	31056	31056	26 178	25 381	25 126	6 080	5 675	5 930
**Butan-1-ol**	33113	31746	31546	26 455	22 989	23 095	6 658	8 757	8 451
**Ethyl acetate**	33223	35971	31250	26316	24272	24876	6907	11699	6374
**Chloroform**	33898	37037	31447	26 667	27 174	24 510	7 231	9 863	6 937
**Cyclohexane**	35336	37313	35971	26 110	34 247	32 895	9 226	3 066	3 076
**Toluen**	33670	31056	31056	26 810	27 933	25 126	6 860	3 123	5 930

Table S2. Physical constants of solvents. The average dipole molecular polarizability α, dielectric constant ε, index of refraction n, functions , F(ε,n) and F(ε+n)+2g(n) of the solvents.

Solvent	α [10^{-24} cm^3]	e	n	E_T (30)		F(ε,n)	F(ε+n)+2g(n)
**Methanol**	3,29	33,00	1,3265	55,5	0,765	0,856	1,301
**Acetonitrile**	4,40	36,64	1,3416	45,6	0,460	0,862	1,327
**Ethanol**	5,41	25,30	1,3594	51,9	0,654	0,817	1,307
**DMSO**	7,30	47,24	1,4773	45,1	0,444	0,842	1,487
**Propan-2-ol**	7,61	20,18	1,3772	48,6	0,552	0,781	1,294
**DMF**	7,81	36,70	1,4305	43,2	0,386	0,836	1,420
**Butan-1-ol**	8,88	17,80	1,3993	49,7	0,586	0,753	1,296
**Ethyl acetate**	8,62	6,08	1,3723	38,1	0,228	0,493	0,999
**Chloroform**	9,50	4,81	1,4429	39,1	0,259	0,372	0,973
**Cyclohexane**	11,00	2,02	1,4262	30,9	0,006	-0,003	0,575
**Toluene**	11,80	2,38	1,4969	33,9	0,099	0,029	0,700

Table S3. Lifetimes (τ) and fractional intensities (f) measured for **TSF**, **TS** and **TB** depending for pH.

Compound	pH	τ_1	f_1	τ_2	f_2
**TSF**	2	0.37±0.10	1.0		
	3	4.03±0.02	1.0		
	4	5.22±0.01	1.0		
	5	5.41±0.04	1.0		
	6	5.43±0.04	1.0		
	7	5.34±0.01	1.0		
	8	5.37±0.01	1.0		
	9	5.25±0.06	1.0		
	10	5.24±0.02	1.0		
	11	5.16±0.05	1.0		
	12	4.97±0.05	1.0		
**TS**	2	1.64±0.06	0.72±0.03	0.29±0.16	0.28±0.03
	3	4.09±0.08	1.0		
	4	4.70±0.07	1.0		
	5	4.87±0.03	1.0		
	6	4.75±0.05	1.0		
	7	4.55±0.11	1.0		
	8	4.59±0.04	1.0		
	9	4.50±0.09	1.0		
	10	4.53±0.03	1.0		
	11	4.34±0.08	1.0		
	12	4.1±0.05	1.0		
**TB**	2	0.10±0.02	0.80±0.05	1.17±0.37	0.20±0.05
	3	0.07±0.03	0.76±0.05	0.65±0.30	0.25±0.05
	4	0.09±0.02	0.90±0.03	1.04±0.37	0.10±0.02
	5	0.07±0.01	0.78±0.01	0.73±0.05	0.21±0.02
	6	0.11±0.02	0.90±0.05	1.14±0.28	0.10±0.05
	7	0.14±0.01	1.0		
	8	0.16±0.01	1.0		
	9	0.15±0.02	1.0		
	10	0.15±0.01	1.0		
	11	0.09±0.02	0.91±0.04	1.29±0.01	0.11±0.04
	12	0.10±0.03	0.83±0.02	1.31±0.12	0.17±0.02

Table S4. Thiadiazole derivativesMICs for 9*Candida*species.

**Organism**	**MIC (µg/ml)**		
	**TB**	**TS**	**TSF**
Candida krusei(Polish isolate)	256	>256	256
Candida fructus(JCM 1513)	128	4	128
Candida fragicola(JCM 1589)	128	32	128
Candidabutyri(JCM 1501)	64	8	128
Candidatropicalis(ATCC 1369)	>256	>256	>256
Candida shehatae(ATCC 22984)	256	64	256
Candidafluviatilis(CBS 6776)	128	32	128
Candidafreyschussi (CBS 3562)	128	128	256
Candida parapsilopsis(DSM 70125)	>256	>256	>256

Table S5. MIC curves interpolations of thiadiazole derivativesagainst*Candida*species.

Control/ MIC	Species	Lag Time (hours)	Max Specific Growth Rate (hours^{-1})	Doubli ng Time (hours)	Max OD	Min OD	R^2
Control	*C. krusei*	3.765	0.034	4.450	1.635	0.057	0.998
TB/MIC	*C. krusei*	5.389	0.069	8.767	1.456	0.044	0.997
TS/MIC	*C. krusei*	n.d.	n.d.	n.d.	n.d.	n.d.	n.d.
TSF/MIC	*C. krusei*	5.869	0.071	9.733	1.574	0.042	0.990
Control	C. *fructus* (ICM 1513)	3.051	0.107	6.446	1.368	0.120	0.999
TB/MIC	C. *fructus* (ICM 1513)	31.858	0.033	20.986	0.533	0.048	0.992
TS/MIC	C. *fructus* (ICM 1513)	27.497	0.029	23.596	0.478	0.022	0.097
TSF/MIC	C. *fructus* (ICM 1513)	23.582	0.026	25.708	0.499	0.018	0.996
Control	*C. fragicola* (ICM 1589)	2.856	0.049	13.976	1.55	0.065	0.999
TB/MIC	*C. fragicola* (ICM 1589)	23.252	0.022	31.131	0.613	0.063	0.998
TS/MIC	*C. fragicola* (ICM 1589)	29.016	0.017	40.526	0.384	0.024	0.985
TSF/MIC	*C. fragicola* (ICM 1589)	12.780	0.038	17.822	1.072	0.044	0.999
Control	*C. butyri* (ICM 1501)	14.981	0.087	7.911	1.569	0.028	0.999
TB/MIC	*C. butyri* (ICM 1501)	18.077	0.045	15.129	1.180	0.042	0.998
TS/MIC	*C. butyri* (ICM 1501)	30.018	0.013	51.816	0.241	0.000	0.970
TSF/MIC	*C. butyri* (ICM 1501)	10.356	0.044	15.465	1.218	0.043	0.998
Control	*C. tropicalis* (ATCC 1369)	3.431	0.187	3.692	1.626	0.048	0.995
TB/MIC	*C. tropicalis* (ATCC 1369)	n.d.	n.d.	n.d.	n.d.	n.d.	n.d.
TS/MIC	*C. tropicalis* (ATCC 1369)	n.d.	n.d.	n.d.	n.d.	n.d.	n.d.
TSF/MIC	*C. tropicalis* (ATCC 1369)	n.d.	n.d.	n.d.	n.d.	n.d.	n.d.
Control	*C. shehatae* (ATCC22984)	1.107	0.071	9.652	1.469	0.037	0.997
TB/MIC	*C. shehatae* (ATCC22984)	12.234	0.056	11.564	0.085	0.064	0.0995

TS/MIC	*C. shehatae* (ATCC22984)	11.223	0.058	10.987	0.087	0.043	0.0996
TSF/MIC	*C. shehatae* (ATCC22984)	26.975	0.047	14.555	0.927	0.041	0.999

Control/ MIC	Species	Lag Time (hours)	Max Specific Growth Rate (hours^{-1})	Doublin g Time (hours)	Max OD	Min OD	R^2
Control	*C. fluviatilis* (CBS 6776)	9.848	0.067	10.267	1.558	0.073	0.998
TB/MIC	*C. fluviatilis* (CBS 6776)	72.941	2.905	20.238	0.921	0.082	0.985
TS/MIC	*C. fluviatilis* (CBS 6776)	27.873	0.024	28.008	0.542	0.042	0.993
TSF/MIC	*C. fluviatilis* (CBS 6776)	99.999	0.322	2.146	0.977	0.038	0.991
Control	C. *freyschussi* (CBS 3562)	10.356	0.065	4.561	1.674	0.065	0.992
TB/MIC	C. *freyschussi* (CBS 3562)	36.112	0.123	5.463	1.431	0.0441	0.995
TS/MIC	C. *freyschussi* (CBS 3562)	37.546	0.124	5.566	1.449	0.042	0.996
TSF/MIC	C. *freyschussi* (CBS 3562)	99.999	0.151	4.583	0.392	0.057	0.992
Control	*C. parapsilopsis* (DSM 70125)	1.000	0.062	11.024	1.370	0.095	0.998
TB/MIC	*C. . parapsilopsis* (DSM 70125)	n.d.	n.d.	n.d.	n.d.	n.d.	n.d.
TS/MIC	*C. . parapsilopsis* (DSM 70125)	n.d.	n.d.	n.d.	n.d.	n.d.	n.d.
TSF/MIC	*C. . parapsilopsis* (DSM 70125)	n.d.	n.d.	n.d.	n.d.	n.d.	n.d.

n.d. not detected
